# Safety and immune responses after a 12-month booster in healthy HIV-uninfected adults in HVTN 100 in South Africa: A randomized double-blind placebo-controlled trial of ALVAC-HIV (vCP2438) and bivalent subtype C gp120/MF59 vaccines

**DOI:** 10.1371/journal.pmed.1003038

**Published:** 2020-02-24

**Authors:** Fatima Laher, Zoe Moodie, Kristen W. Cohen, Nicole Grunenberg, Linda-Gail Bekker, Mary Allen, Nicole Frahm, Nicole L. Yates, Lynn Morris, Mookho Malahleha, Kathryn Mngadi, Brodie Daniels, Craig Innes, Kevin Saunders, Shannon Grant, Chenchen Yu, Peter B. Gilbert, Sanjay Phogat, Carlos A. DiazGranados, Marguerite Koutsoukos, Olivier Van Der Meeren, Carter Bentley, Nonhlanhla N. Mkhize, Michael N. Pensiero, Vijay L. Mehra, James G. Kublin, Lawrence Corey, David C. Montefiori, Glenda E. Gray, M. Juliana McElrath, Georgia D. Tomaras

**Affiliations:** 1 Perinatal HIV Research Unit, Faculty of Health Sciences, University of the Witwatersrand, Johannesburg, South Africa; 2 Vaccine and Infectious Disease Division, Fred Hutchinson Cancer Research Center, Seattle, Washington, United States of America; 3 Desmond Tutu HIV Centre, University of Cape Town, Cape Town, South Africa; 4 Vaccine Research Program, Division of AIDS, National Institute of Allergy and Infectious Diseases, National Institutes of Health, Bethesda, Maryland, United States of America; 5 Departments of Surgery and Immunology, Duke Human Vaccine Institute, Durham, North Carolina, United States of America; 6 National Institute for Communicable Diseases, National Health Laboratory Service, Johannesburg, South Africa; 7 Faculty of Health Sciences, University of the Witwatersrand, Johannesburg, South Africa; 8 Setshaba Research Centre, Soshanguve, South Africa; 9 Centre for the AIDS Programme of Research in South Africa, Durban, South Africa; 10 South African Medical Research Council, Durban, South Africa; 11 Aurum Institute, Klerksdorp Research Centre, Klerksdorp, South Africa; 12 Sanofi Pasteur, Swiftwater, Pennsylvania, United States of America; 13 GSK Vaccines, Rixensart, Belgium; Faculty of Tropical Medicine, Mahidol University, THAILAND

## Abstract

**Background:**

HVTN 100 evaluated the safety and immunogenicity of an HIV subtype C pox-protein vaccine regimen, investigating a 12-month booster to extend vaccine-induced immune responses.

**Methods and findings:**

A phase 1–2 randomized double-blind placebo-controlled trial enrolled 252 participants (210 vaccine/42 placebo; median age 23 years; 43% female) between 9 February 2015 and 26 May 2015. Vaccine recipients received ALVAC-HIV (vCP2438) alone at months 0 and 1 and with bivalent subtype C gp120/MF59 at months 3, 6, and 12. Antibody (IgG, IgG3 binding, and neutralizing) and CD4+ T-cell (expressing interferon-gamma, interleukin-2, and CD40 ligand) responses were evaluated at month 6.5 for all participants and at months 12, 12.5, and 18 for a randomly selected subset. The primary analysis compared IgG binding antibody (bAb) responses and CD4+ T-cell responses to 3 vaccine-matched antigens at peak (month 6.5 versus 12.5) and durability (month 12 versus 18) timepoints; IgG responses to CaseA2_gp70_V1V2.B, a primary correlate of risk in RV144, were also compared at these same timepoints. Secondary and exploratory analyses compared IgG3 bAb responses, IgG bAb breadth scores, neutralizing antibody (nAb) responses, antibody-dependent cellular phagocytosis, CD4+ polyfunctionality responses, and CD4+ memory sub-population responses at the same timepoints. Vaccines were generally safe and well tolerated. During the study, there were 2 deaths (both in the vaccine group and both unrelated to study products). Ten participants became HIV-infected during the trial, 7% (3/42) of placebo recipients and 3% (7/210) of vaccine recipients. All 8 serious adverse events were unrelated to study products. Less waning of immune responses was seen after the fifth vaccination than after the fourth, with higher antibody and cellular response rates at month 18 than at month 12: IgG bAb response rates to 1086.C V1V2, 21.0% versus 9.7% (difference = 11.3%, 95% CI = 0.6%–22.0%, *P* = 0.039), and ZM96.C V1V2, 21.0% versus 6.5% (difference = 14.5%, 95% CI = 4.1%–24.9%, *P* = 0.004). IgG bAb response rates to all 4 primary V1V2 antigens were higher 2 weeks after the fifth vaccination than 2 weeks after the fourth vaccination: 87.7% versus 75.4% (difference = 12.3%, 95% CI = 1.7%–22.9%, *P* = 0.022) for 1086.C V1V2, 86.0% versus 63.2% (difference = 22.8%, 95% CI = 9.1%–36.5%, *P* = 0.001) for TV1c8.2.C V1V2, 67.7% versus 44.6% (difference = 23.1%, 95% CI = 10.4%–35.7%, *P* < 0.001) for ZM96.C V1V2, and 81.5% versus 60.0% (difference = 21.5%, 95% CI = 7.6%–35.5%, *P* = 0.002) for CaseA2_gp70_V1V2.B. IgG bAb response rates to the 3 primary vaccine-matched gp120 antigens were all above 90% at both peak timepoints, with no significant differences seen, except a higher response rate to ZM96.C gp120 at month 18 versus month 12: 64.5% versus 1.6% (difference = 62.9%, 95% CI = 49.3%–76.5%, *P* < 0.001). CD4+ T-cell response rates were higher at month 18 than month 12 for all 3 primary vaccine-matched antigens: 47.3% versus 29.1% (difference = 18.2%, 95% CI = 2.9%–33.4%, *P* = 0.021) for 1086.C, 61.8% versus 38.2% (difference = 23.6%, 95% CI = 9.5%–37.8%, *P* = 0.001) for TV1.C, and 63.6% versus 41.8% (difference = 21.8%, 95% CI = 5.1%–38.5%, *P* = 0.007) for ZM96.C, with no significant differences seen at the peak timepoints. Limitations were that higher doses of gp120 were not evaluated, this study was not designed to investigate HIV prevention efficacy, and the clinical significance of the observed immunological effects is uncertain.

**Conclusions:**

In this study, a 12-month booster of subtype C pox-protein vaccines restored immune responses, and slowed response decay compared to the 6-month vaccination.

**Trial registration:**

ClinicalTrials.gov NCT02404311.

South African National Clinical Trials Registry (SANCTR number: DOH--27-0215-4796).

## Abstract 2

Fatima Laher and co-authors report on safety and immune responses in a randomized trial of a candidate HIV vaccine incorporating a "booster" vaccination.

## Author summary

**Why was this study done?:**

- The RV144 vaccine trial, which tested a pox-protein regimen designed against HIV subtypes B and E, showed modest efficacy in preventing HIV acquisition. However, efficacy and vaccine-induced immune responses declined considerably after the first year.
- Our clinical trial, HVTN 100, investigated whether adding a booster at month 12 to a similar vaccine regimen, tailored against subtype C HIV, would prolong immune responses, and also evaluated its safety.

**What did the researchers do and find?:**

- We gave intramuscular injections of ALVAC-HIV (vCP2438) alone at months 0 and 1 and with bivalent subtype C gp120/MF59 at months 3, 6, and 12 to 210 vaccine recipients. We administered placebo to 42 participants.
- We tested antibody (IgG, IgG3 binding, antibody-dependent cellular phagocytosis, and neutralizing) and cellular (T cells expressing interferon-gamma, interleukin-2, or CD40 ligand) immune responses at month 6.5 in all participants and at months 12, 12.5, and 18 in a randomly chosen subset. We evaluated safety for 18 months.
- Vaccines were generally safe and well tolerated. Antibody and cellular response rates were higher after the 12-month booster than after the 6-month vaccination.

**What do these findings mean?:**

- Adding a booster osf subtype C pox-protein vaccines at month 12 can improve the durability of vaccine-induced immune responses.
- The ongoing phase 2b–3 trial HVTN 702 was designed to evaluate the efficacy of this regimen with month 12 and month 18 boosters.

## Introduction

The goal of a preventative HIV vaccine is to induce durable immune responses that can protect against HIV acquisition. Durability of a prevention intervention reduces health system burden and cost. Durability was a critical challenge in the RV144 trial, which administered the only vaccine regimen to date to show any preventative efficacy against HIV-1 acquisition [1–3]. With a 4-dose pox-protein heterologous prime–boost regimen administered over 6 months, vaccine efficacy in the RV144 trial was 60.5% at 1 year but waned to 31.2% at 3.5 years [4]. Similarly, antibody response magnitude and quality decayed significantly over the same time frame, with IgG3 antibodies to HIV envelope decaying earlier than other subclasses. IgG and IgG3 binding antibodies (bAbs) to the HIV envelope and IgG3 bAbs to the V1V2 region were among the immune responses that correlated with decreased HIV-1 acquisition risk [5].

Extending antibody persistence and improving the quality of responses could be approached through multiple plausible strategies, including altering immunogen design, adjuvant selection, dosing intervals, and administration routes. Currently, several clinical trials are exploring the use of various adjuvants and additional booster doses to extend the longevity of vaccine-induced immune responses. Both non-human primate and human studies utilizing other pox-protein boosters have demonstrated that, although immune responses decline after completion of the primary vaccination series, a booster can restore antibody responses [6].

The HIV Vaccine Trials Network (HVTN) 100 clinical trial investigated a prime–boost vaccine regimen designed against subtype C HIV, and it aimed to increase the magnitude and duration of vaccine-elicited immune responses beyond those observed in the RV144 trial. The regimen had 2 constituents. The first constituent was ALVAC-HIV, a pox vector vaccine with subtype C inserts. The second constituent was glycoprotein 120 (gp120), a protein vaccine that was administered with the MF59 adjuvant, instead of the alum adjuvant used in the RV144 trial. HVTN 100 also added a pox-protein booster (a fifth vaccination) at month 12. By 2 weeks after the month 6 vaccination, all HVTN 100 vaccine recipients developed IgG bAbs with significantly higher titers than to the corresponding antigens in the RV144 trial [7]. Here we present the safety profile and cellular and antibody responses, including the effects of the 12-month booster, up to month 18.

## Methods

### Study design, population, products, and procedures

The HVTN 100 study design, eligibility criteria, participants and their baseline characteristics, randomization by computer-generated block-randomized sequences, masking of participants and staff, study products, and trial site locations have been described in detail elsewhere [7]. All participants gave written informed consent in English or their local language (Setswana, Sotho, Xhosa, or Zulu). Participant baseline characteristics of the intention-to-treat (i.e., full study), per-protocol, and durability subset cohorts are shown in S1 Table. HVTN 100 is, in summary, a phase 1–2randomized double-blind placebo-controlled trial of 252 healthy HIV-uninfected 18- to 40-year-old participants at 6 sites in South Africa. The investigational products were ALVAC-HIV (vCP2438) and MF59-adjuvanted bivalent subtype C gp120. ALVAC-HIV (vCP2438) (viral titer nominal dose of 10^7^ 50% cell culture infectious dose) expressed the envelope gp120 of the subtype C ZM96.C strain with the gp41 transmembrane sequence of the subtype B LAI strain, as well as *gag* and *protease* from the subtype B LAI strain. Bivalent subtype C gp120 was a combination of 100 μg each of the subtype C envelope gp120 of the TV1.C and 1086.C strains. Placebo for ALVAC-HIV was a mixture of virus stabilizer and freeze-drying medium reconstituted with 0.4% NaCl, and placebo for the bivalent subtype C gp120 was sodium chloride for injection. Participants were randomized in a 5:1 vaccine:placebo ratio to receive ALVAC-HIV at months 0 and 1 followed by 3 administrations of ALVAC-HIV along with bivalent subtype C gp120/MF59 at months 3, 6, and 12, or to receive placebo throughout, all by intramuscular injection. The protocol specified ALVAC-HIV or placebo to be administered in the left deltoid and bivalent subtype C gp120/MF59 or placebo to be administered in the right deltoid. Participants were followed for 18 months after enrollment.

A copy of the protocol is available online at https://atlas.scharp.org/cpas/project/HVTN%20Public%20Data/HVTN%20100/begin.view.

### Safety measures

Standard safety laboratory testing included hematology, serum chemistry, and urinalysis, which were obtained at baseline (during screening) and at each 2-week post-vaccination visit plus a month 15 visit (except urinalysis, collected at 2 weeks after the first, fourth, and fifth vaccination). Participants were observed for 30 minutes after vaccinations and recorded solicited local and systemic symptoms (reactogenicity) for 3 days after each vaccination. Adverse events (AEs) were recorded until 30 days after each vaccination, except for AEs leading to early participant withdrawal or early product discontinuation and serious AEs, which were recorded throughout the trial. AEs were coded using the Medical Dictionary for Regulatory Activities (MedDRA), version 21.1, and severity was graded using version 2.0 of the DAIDS Table for Grading the Severity of Adult and Pediatric Adverse Events (November 2014). Safety reviews were conducted by the protocol safety review team and the National Institute of Allergy and Infectious Diseases (NIAID) Data and Safety Monitoring Board (DSMB).

### Laboratory assays

Laboratory assays [8–11] were performed blinded in HVTN laboratories. Cellular and antibody immune responses were evaluated at months 6.5, 12, 12.5, and 18. The frequency and magnitude of IgG and IgG3 bAb responses were measured by the HIV-1 binding antibody multiplex assay in serum specimens as previously described [5,12,13]. The ability of vaccine-induced antibodies to engage Fc-mediated antibody-dependent cellular phagocytosis (ADCP) was measured using previously described methods [14]. The neutralizing antibody (nAb) assay was performed with Env-pseudotyped viruses in TZM-bl cells as previously described [10]. Intracellular cytokine staining (ICS) was performed to measure HIV-specific T cells expressing IFN-γ, IL-2, or CD40L [15] using a 16-color assay. Details on the antigens used in laboratory assays are shown in S2 Table.

### Approvals

The research ethics committees of the University of the Witwatersrand, the University of Cape Town, the University of KwaZulu-Natal, and the South African Medical Research Council approved the study. The trial was overseen by the NIAID DSMB. HVTN 100 was registered with the South African National Clinical Trials Register (SANCTR number: DOH—27-0215-4796) and ClinicalTrials.gov (NCT02404311). The first participant was enrolled on 9 February 2015. The trial registration process was initiated prior to this date, and was achieved on 9 February 2015 at SANCTR and on 31 March 2015 at ClinicalTrials.gov.

### Statistical analysis

The primary objectives of this study were to compare IgG responses to vaccine-matched gp120 and V1V2 antigens (ZM96.C, 1086.C, TV1c8.2.C) and CaseA2_gp70_V1V2.B and CD4+ T-cell responses to vaccine-matched peptide pools (Env ZM96.C, 1086.C, TV1c8.2.C) at the month 6.5 versus 12.5 peak timepoints (2 weeks after the fourth and fifth vaccination) and at the month 12 versus 18 durability timepoints (6 months after the fourth and fifth vaccination). The secondary objectives were to compare IgG3 responses to the same antigens at the same timepoints as IgG, nAb responses to TV1c8.2.C and MW965.26.C, and ADCP responses to 1086.C gp140 at the same peak and durability timepoints. CD4+ memory sub-populations and CD4+ polyfunctionality responses (as defined below) to vaccine-matched peptide pools were assessed as exploratory objectives.

All randomized participants were included in the safety analyses. The number and percentage of participants experiencing each AE or reactogenicity symptom were tabulated by severity.

Immune responses at month 6.5 were analyzed in the per-protocol (those who received the first 4 scheduled vaccinations) cohort of 185 vaccine recipients and 37 placebo recipients, except ADCP responses, which were measured on the durability subset cohort (described next) at all timepoints. Immune responses at months 12, 12.5, and 18 were analyzed on the durability subset cohort, a subset of 70 vaccine recipients and 5 placebo recipients, randomly selected from among per-protocol cohort participants who had month 6.5 samples available; subset selection was stratified by sex to match the sex ratio observed in HVTN 100.

McNemar’s test and Wilcoxon signed rank test were used to compare response rates and magnitudes (overall and among responders), respectively, between 2 timepoints. Two-sided 95% confidence intervals (CIs) were computed using the Wilson method [16] for the positive response rates, using the central CIs from McNemar’s test for the difference in positive response rates between 2 timepoints, and using the normal approximation for the mean of the log-transformed magnitudes. The magnitude–breadth curve, with the area under the curve (AUC) computed as a summary measure, was used to describe the magnitude and breadth across a panel of antigens [17]. The AUC, or “breadth score,” represents the area under the magnitude–breadth curve for an individual vaccine recipient, calculated as the average of the log_10_ (MFI-blank) over the panel of antigens, where each antigen receives equal weight. Antigen-specific T-cell subsets were analyzed by COMPASS (Combinatorial Polyfunctionality Analysis of Antigen-Specific T-cell Subsets), where the functionality score is defined as the estimated proportion of Env-specific subsets detected among all possible subsets, while the polyfunctionality score is similar but weights the different subsets by their degree of functionality, favoring subsets with higher degrees of functions [18]. COMPASS posterior probabilities were reported for all observed 6-function CD4+ T-cell subsets (interferon-gamma, interleukin-2, tumor necrosis factor alpha, CD40 ligand, interleukin-4, and granzyme B) over time.

All tests were 2-sided, and differences were considered statistically significant if *P* value < 0.05. Multiplicity adjustments were not made. Statistical analyses were performed using SAS (version 9.4, SAS Institute, Cary, NC) and R statistical software (version 3.3.2, R Foundation for Statistical Computing, Vienna, Austria). We followed CONSORT guidelines for reporting clinical trials (S1 CONSORT Checklist).

## Results

### Study population

**Fig 1:**
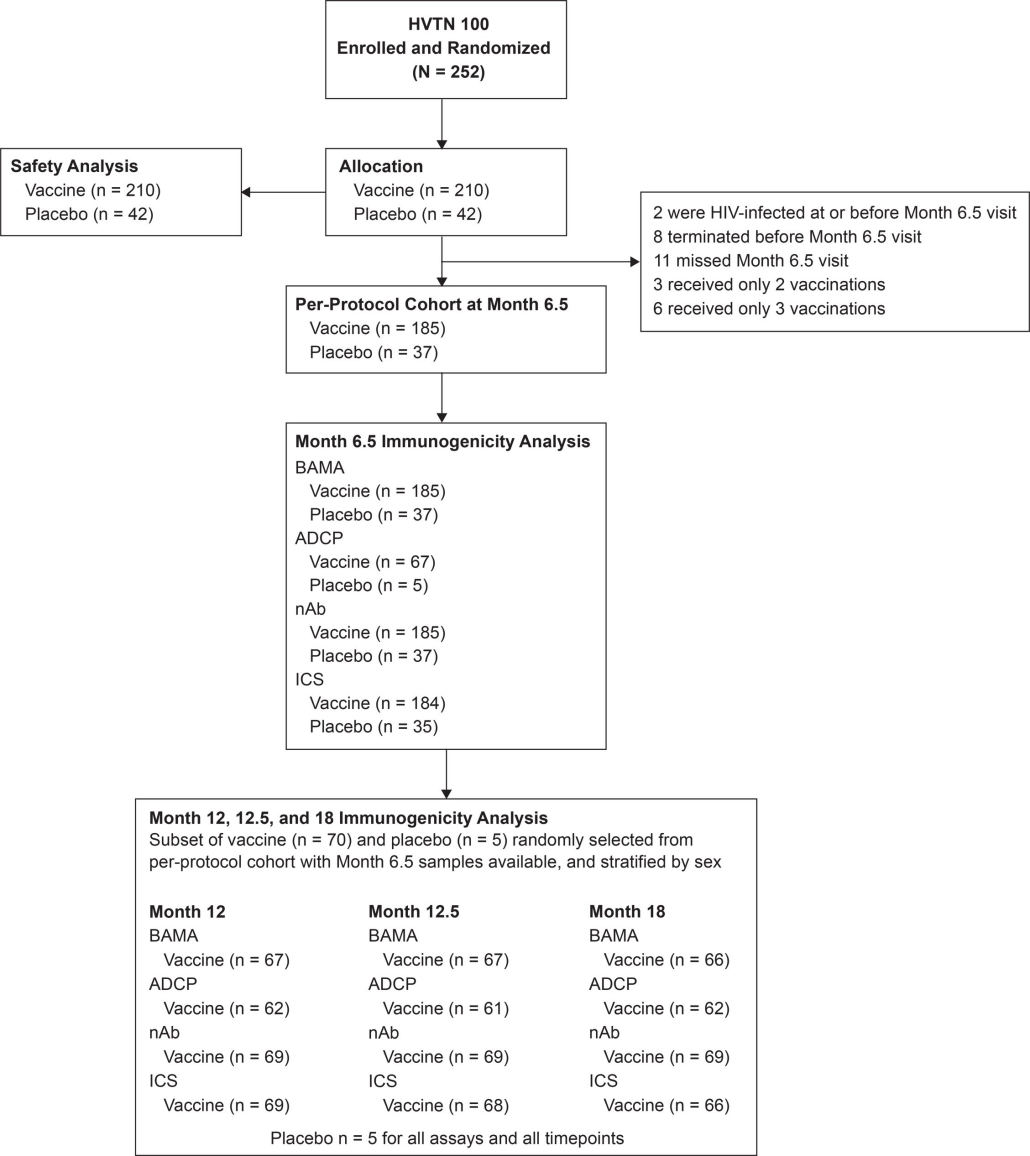
CONSORT flow diagram of the HVTN 100 trial. Per-protocol cohort at month 6.5 is defined as receipt of the first 4 scheduled vaccinations. ADCP, antibody-dependent cellular phagocytosis; BAMA, binding antibody multiplex assay; ICS, intracellular cytokine staining; nAb, neutralizing antibody.

Of 252 participants enrolled in HVTN 100 between 9 February 2015 and 26 May 2015, 210 were randomized to vaccine and 42 to placebo. Median age was 23 years (interquartile range 21–27), and 109 (43%) were women. By month 6.5 (2 weeks after the fourth vaccination), the per-protocol cohort included 185 vaccine recipients and 37 placebo recipients (Fig 1). Of all 252 participants, 214 (85%) received all 5 vaccinations: 90% (38/42) in the placebo group and 84% (176/210) in the vaccine group.

### Safety and reactogenicity

**Fig 2:**
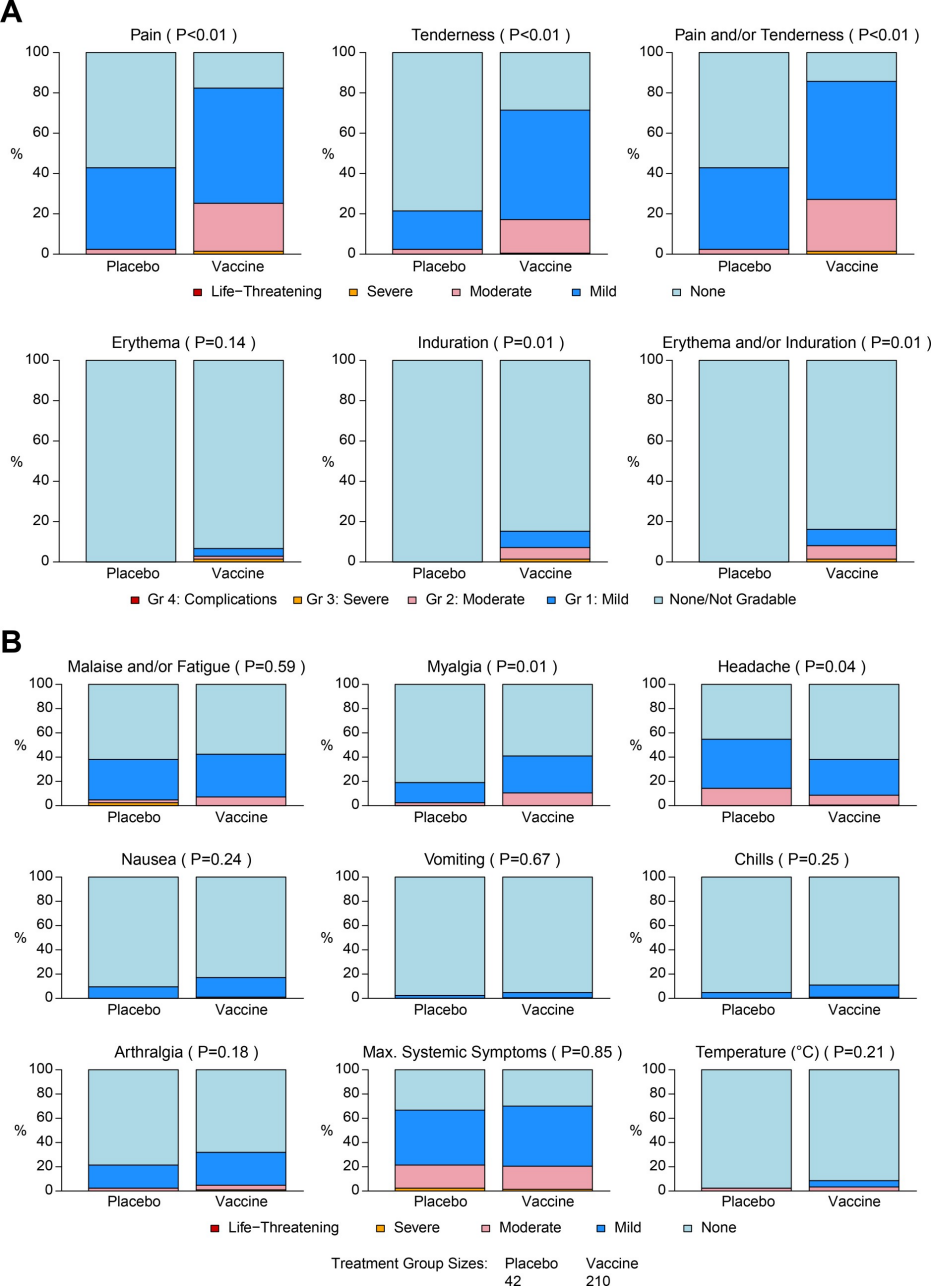
Stacked bar charts of maximum local and systemic reactogenicity over all vaccinations. Local (A) and systemic (B) reactogenicity events. *P* values indicate differences between vaccine and placebo groups. Reactogenicity event grading color-code: red = life-threatening, orange = severe, pink = moderate, dark blue = mild, light blue = none.

Vaccinations were generally safe and well tolerated. Grade 3 pain and/or tenderness was reported by 1.4% (3/210) of vaccine recipients, Grade 3 erythema and/or induration was reported by 1.4% (3/210) of vaccine recipients, and maximum systemic reactogenicity (headache, arthralgia) of Grade 3 was reported by 1.4% (3/210) of vaccine recipients. No Grade 4 local or systemic reactogenicities were reported (Fig 2). All Grade 3 systemic reactogenicity events and local pain and/or tenderness events resolved within 3 days of vaccination, except for 1 Grade 3 arthralgia event, which occurred after the first vaccination and resolved within 9 days. One vaccine recipient reported Grade 3 erythema and induration on the right deltoid beginning on day 3 after the fifth vaccination: antibiotic, anti-inflammatory, and analgesic medications were taken for 3 days, and there was resolution by day 5. Another vaccine recipient reported Grade 3 erythema and induration in the right deltoid beginning on day 2 after the fourth vaccination that resolved within 1 day (induration) and 4 days (erythema) of onset (3 and 6 days post-vaccination, respectively). Antibiotics, analgesics, and antihistamines were prescribed, and the participant was discontinued from further vaccinations according to protocol but remained in follow-up. A third participant experienced Grade 3 erythema and induration in the left deltoid, with onset on day 0 after the fifth vaccination and resolving by day 3. Antihistamine, oral steroid, and analgesic/anti-inflammatory medications were taken. In all 3 participants with Grade 3 erythema/induration, the needle used for injection was <1.5 inches long, consistent with weight-based guidance for needle length choice. Pain, tenderness, induration, myalgia, and headache were significantly higher in the vaccine group than in the placebo group (*P* = 0.04 for headache, all other *P* ≤ 0.01; Fig 2), but these symptoms were mostly mild to moderate and of short duration.

Unsolicited AEs were reported by 71.8% (181/252) of participants, with no significant difference in AE reporting between the placebo and vaccine groups (81.0% [34/42] versus 70.0% [147/210], respectively, *P* = 0.153). Eleven participants experienced 14 AEs deemed by the investigator to be attributed to the study products, including 3/42 (7.1%) placebo recipients (headache, diarrhea, injection site pruritus, and oral paresthesia) and 8/210 (3.8%) vaccine recipients (abdominal pain, gastritis, injection site pruritis in 2 participants, injection site nodule, lymphadenopathy in 2 participants, dizziness, decreased neutrophil count, and generalized pruritus): all were mild or moderate, and transient. There were 3 potentially life-threatening AEs, all deemed to be unrelated to study products: raised aspartate aminotransferase (asymptomatic with an alternative etiology, resolved within 1 week), depression (ongoing at end of study), and subdural hematoma. There were 2 deaths, both in vaccine recipients, and both deemed unrelated to study product (multiple injuries with subdural hematoma, and suicide). There were 8 serious AEs reported by 6 participants, and all were deemed unrelated to the study products.

Before completing the trial, 16.7% (7/42) of placebo recipients and 12.4% (26/210) of vaccine recipients were terminated from the study, with reasons including HIV infection, inability to contact participant, persistent non-adherence to study schedule, participant refusal, relocation, death, and investigator decision. Vaccinations were discontinued in 7.1% (3/42) of placebo recipients and 8.1% (17/210) of vaccine recipients. In 7 participants, AEs led to early withdrawal/termination from study or discontinuation of vaccination: 1 placebo recipient (Grade 1 vomiting on day 1 after the first vaccination) and 6 vaccine recipients (2 psychiatric diagnoses unrelated to study product, death from multiple injuries, death from suicide, Grade 3 hypertension unrelated to study product, and Grade 3 erythema and induration reaction).

One placebo recipient and 1 vaccine recipient became pregnant; both had full-term live births with no congenital abnormalities reported.

HVTN 100 included participants who were deemed to be at low risk for HIV acquisition, as per HVTN low risk guidance for South African sites and investigator assessment: 4% (10/252) became HIV-infected during the trial, 7.1% (3/42) in the placebo group and 3.3% (7/210) in the vaccine group. All HIV diagnoses were made at a timepoint that was beyond 3 months since enrollment, and all participants who acquired HIV received at least 3 vaccinations.

### Vaccine-induced seroreactivity

At the end of trial (18 months after enrollment and 6 months after the final vaccination), 1/202 (0.5%, 95% CI = 0.1%–2.8%) vaccine recipient had vaccine-induced seroreactivity by BioRad Multispot HIV-1/HIV-2 Rapid Test (an assay that has since become obsolete).

### bAb responses

**Fig 3:**
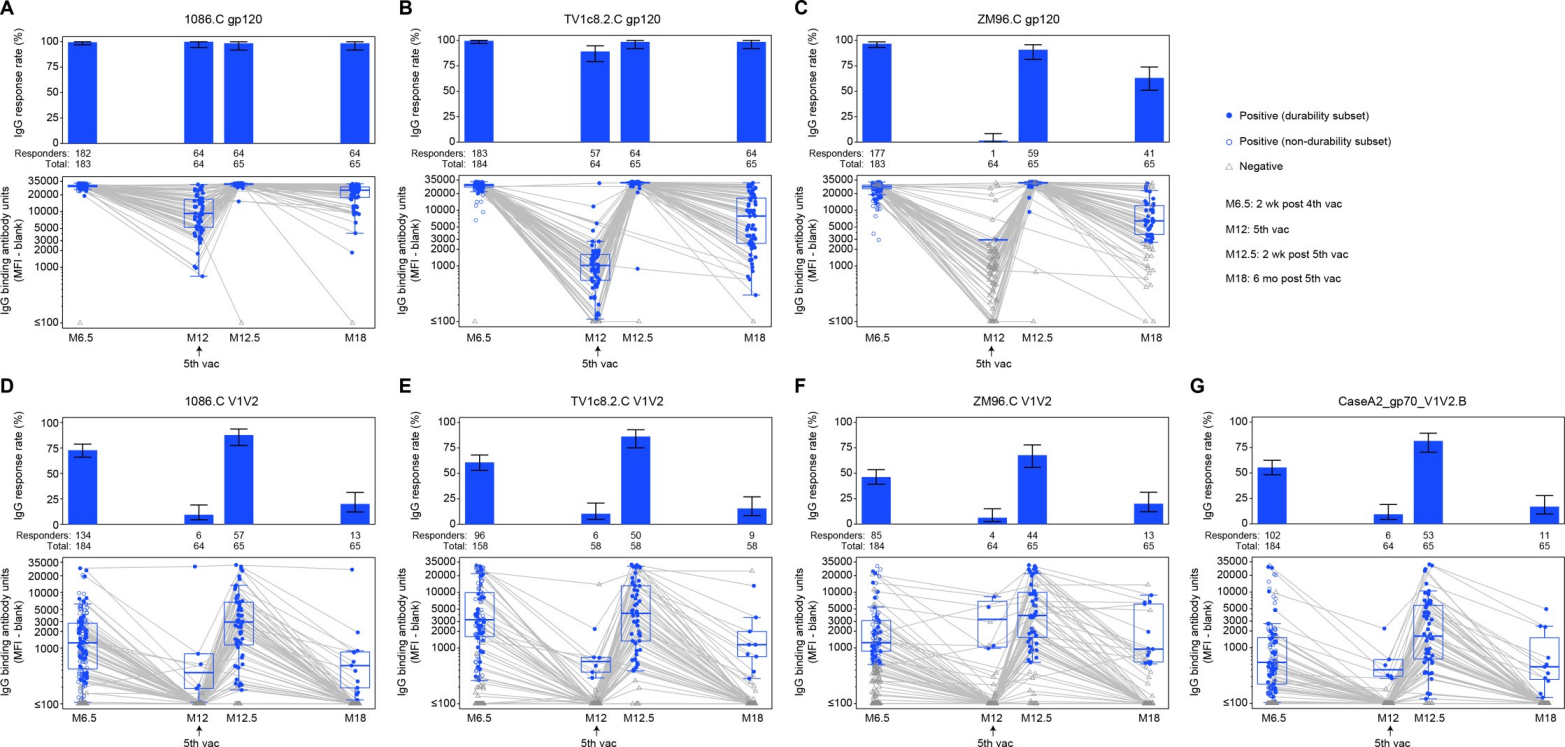
Summary of response rates and magnitudes of IgG binding antibodies to gp120 and V1V2 antigens among per-protocol vaccine recipients of HVTN 100. Responses to gp120 (A–C) and V1V2 antigens (D–G). Bar charts show response rates with 2-sided 95% CIs. Boxplots show magnitude as log_10_ (MFI-blank) responses to individual gp120 and V1V2 antigens and are based on positive responders, shown as solid blue circles; negative responders are shown as grey triangles. Grey lines connect the response magnitude of each per-protocol vaccine recipient over time. M[number], month [number]; vac, vaccination.

We previously reported that the subtype C ALVAC/protein boost regimen elicited high antibody IgG titers at month 6.5 [7]. Here we report that IgG bAb response rates and magnitudes were high 2 weeks after the fourth and fifth vaccinations (months 6.5 and 12.5) (Fig 3). Response rates to the vaccine-matched gp120 antigens in the protein (1086.C and TV1.C) remained high (>90%) at the month 12 and 18 durability timepoints, but the magnitude among positive responders to TV1.C, as well as the response rates and magnitudes among positive responders to ZM96.C gp120 and the V1V2 antigens, were markedly decreased at months 12 and 18 (Fig 3). Evaluating paired samples, IgG bAb response rates were significantly higher at month 18 compared to month 12: 21.0% versus 9.7% (difference = 11.3%, 95% CI = 0.6%–22.0%, *P* = 0.039) for 1086.C V1V2 and 21.0% versus 6.5% (difference = 14.5%, 95% CI = 4.1%–24.9%, *P* = 0.004) for ZM96.C V1V2 (Fig 3A–3C; S3 Table). IgG bAb response rates to all 4 primary V1V2 antigens were higher 2 weeks after the fifth vaccination than 2 weeks after the fourth vaccination: 87.7% versus 75.4% (difference = 12.3%, 95% CI = 1.7%–22.9%, *P* = 0.022) for 1086.C V1V2, 86.0% versus 63.2% (difference = 22.8%, 95% CI = 9.1%–36.5%, *P* = 0.001) for TV1c8.2.C V1V2, 67.7% versus 44.6% (difference = 23.1%, 95% CI = 10.4%–35.7%, *P* < 0.001) for ZM96.C V1V2, and 81.5% versus 60.0% (difference = 21.5%, 95% CI = 7.6%–35.5%, *P* = 0.002) for CaseA2_gp70_V1V2.B (S3 Table).

IgG bAb response rates to the 3 primary vaccine-matched gp120 antigens were all above 90% at all 4 timepoints, with no significant differences seen apart from a higher response rate to ZM96.C gp120 at month 18 than month 12: 64.5% versus 1.6% (difference = 62.9%, 95% CI = 49.3%–76.5%, *P* < 0.001) (S3 Table). The response magnitude overall and among positive responders to all 7 primary antigens was significantly higher at month 12.5 than at month 6.5 (all *P* < 0.001 except *P* = 0.02 for TV1c8.2.C V1V2). Higher overall response magnitudes were seen at month 18 versus month 12 for all 7 primary antigens (all *P* < 0.001 except *P* = 0.016 for 1086.C V1V2, *P* = 0.009 for TV1c8.2.C V1V2, and *P* = 0.011 for CaseA2_gp70_V1V2.B), but response magnitudes among positive responders were only higher for 1086.C gp120 and TV1c8.2.C gp120 (both *P* < 0.001; estimates and 95% CIs in S3 Table).

In a secondary analysis, we evaluated the breadth of antibody responses utilizing pre-specified panels to examine global coverage [13].

**Fig 4:**
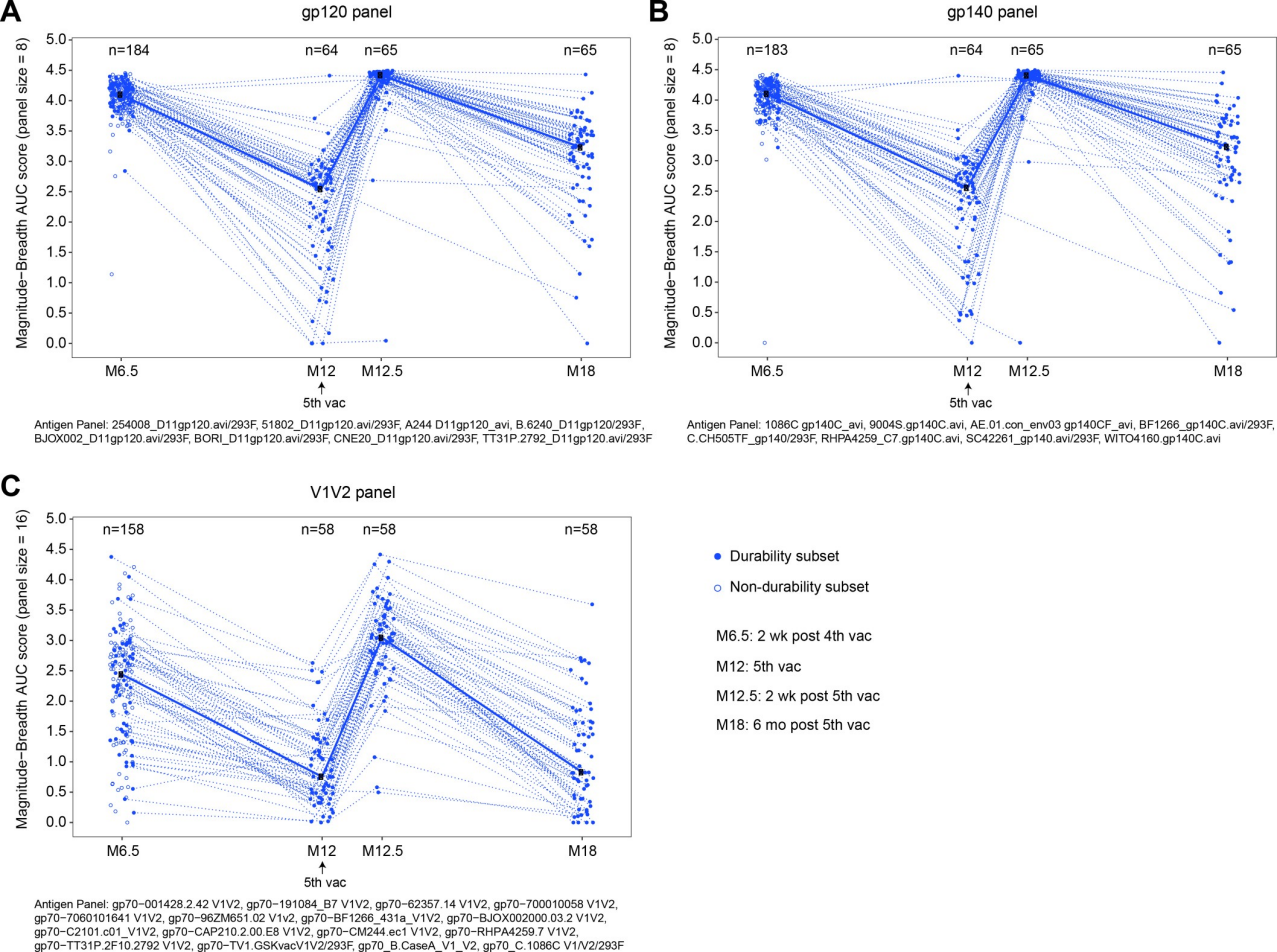
Breadth of IgG binding antibody responses to gp120, gp140, and V1V2 antigens among per-protocol vaccine recipients of HVTN 100. Responses to gp120 (A), gp140 (B), and V1V2 (C) antigens. Each point represents the area under the magnitude–breadth curve for an individual vaccine recipient, calculated as the average of the log_10_ (MFI-blank) over the panel of antigens, where antigens are listed in the footnote below each plot. Dashed lines connect the area under the magnitude–breadth curve of each per-protocol vaccine recipient over time. AUC, area under the curve; M[number], month [number]; vac, vaccination.

The breadth of IgG responses amongst vaccine recipients to the gp120, gp140, and V1V2 antigen panels was significantly higher at month 12.5 than month 6.5 (all *P* < 0.001) and also higher at month 18 than at month 12 (*P* < 0.001, *P* < 0.001, and *P* = 0.001, respectively) (Fig 4), indicating that IgG envelope breadth was boosted. In addition to conformational V1V2 antibody responses, linear V2 IgG responses, a correlate of decreased HIV-1 risk in the RV144 trial [19], were also elicited in HVTN 100 [20].

IgG3 bAb response rates were significantly higher at month 6.5 than at month 12.5 for 1086.C gp120 and TV1c8.2.C gp120 (*P* = 0.016 and *P* = 0.011, with estimates and 95% CIs in S4 Table), and were similar at month 18 compared to month 12 for all 3 vaccine-matched gp120 antigens (*P* = 0.75, 1.00, and 0.63, with estimates and 95% CIs in S4 Table; S1 Fig) and for all 4 V1V2 antigens at all timepoints (vaccine-matched antigens shown in S1 Fig) (all *P >* 0.50). The response magnitude overall and among positive responders to 1086.C gp120 was significantly higher at month 6.5 than at month 12.5 (both *P* < 0.001, with estimates and 95% CIs in S4 Table) and significantly higher at month 12.5 than month 6.5 for ZM96.C gp120 (*P* < 0.001 overall and *P* = 0.027 among positive responders, with estimates and 95% CIs in S4 Table). Response magnitudes did not differ for other antigens or between months 12 and 18 (all *P >* 0.16, with estimates and 95% CIs in S4 Table), except higher overall magnitudes for TV1c8.2.C gp120 at month 6.5 versus month 12.5 (*P* = 0.009) and at month 18 versus month 12 (*P* = 0.049). These results indicate that IgG3 responses, unlike those for IgG, were generally not boosted, as can also be seen by the magnitude–breadth AUC scores across the gp120, gp140, and V1V2 panels in S2 Fig.

### ADCP responses

**Fig 5:**
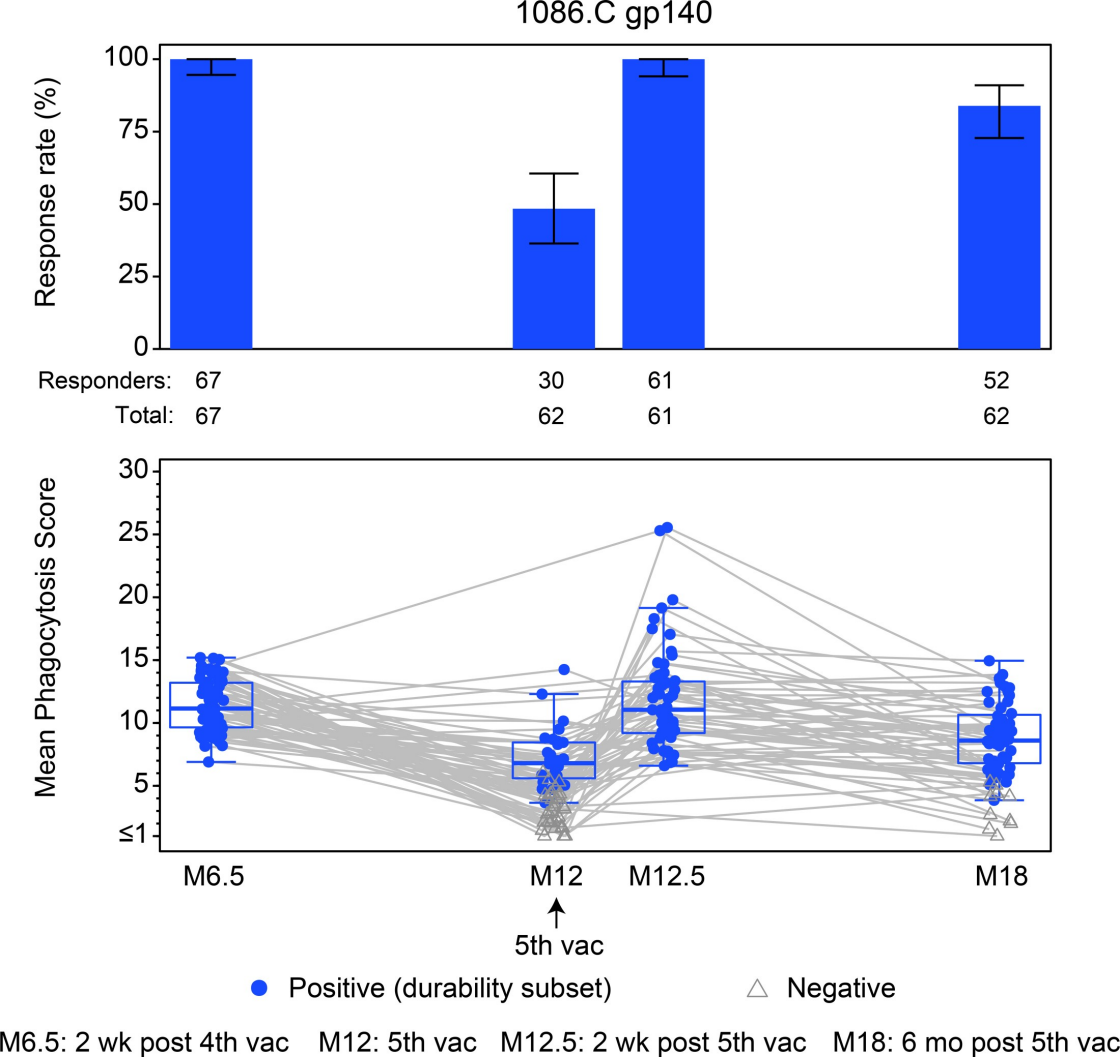
Summary of response rates and magnitudes of antibody-dependent cellular phagocytosis to 1086.C gp140 among per-protocol vaccine recipients of HVTN 100. Bar charts show response rates with 2-sided 95% CIs. Boxplots show magnitude as mean phagocytosis scores to 1086.C gp140 and are based on positive responders, shown as solid blue circles; negative responders are shown as grey triangles. Grey lines connect the response magnitude of each per-protocol vaccine recipient over time. M[number], month [number]; vac, vaccination.

We also examined antibody Fc effector function ADCP, a response that was associated with antibody responses that correlated with decreased HIV-1 acquisition risk in RV144 [21,22] and that correlated with decreased HIV-1 risk in HVTN 505 [23]. The ADCP response rate against 1086.C gp140 was 100.0% (95% CI = 94.6%–100.0%) among per-protocol vaccine recipients at 2 weeks after the fourth vaccination (month 6.5), declined to 48.4% (95% CI = 36.4%–60.6%) after 6 months (month 12), was boosted back to 100.0% (95% CI = 94.1%–100%) at 2 weeks after the fifth vaccination (month 12.5), and then declined to 83.9% (95% CI = 72.8%–91.0%) 6 months later (month 18) (Fig 5). As expected, the ADCP magnitude was highest 2 weeks after the fourth and fifth vaccinations; there was no statistically significant difference in magnitude between these measured peak timepoints (*P* = 0.961). Notably, when comparing the 6-month timepoints after the fifth and fourth vaccinations, the ADCP response rate and the ADCP response magnitude overall and among positive responders at month 18 were significantly higher than at month 12, suggesting that the additional booster improved the durability (*P*_rate_ < 0.001, *P*_magnitude overall_ < 0.001, *P*_magnitude among responders_ = 0.001, with estimates and 95% CIs in S4 Table). The response rate for placebo recipients at each timepoint was 0%.

### Cellular immune responses

We evaluated vaccine-specific T-cell responses to the vector insert (ZM96) as well as to the gp120 proteins (TV1 and 1086) included in the boosts.

**Fig 6:**
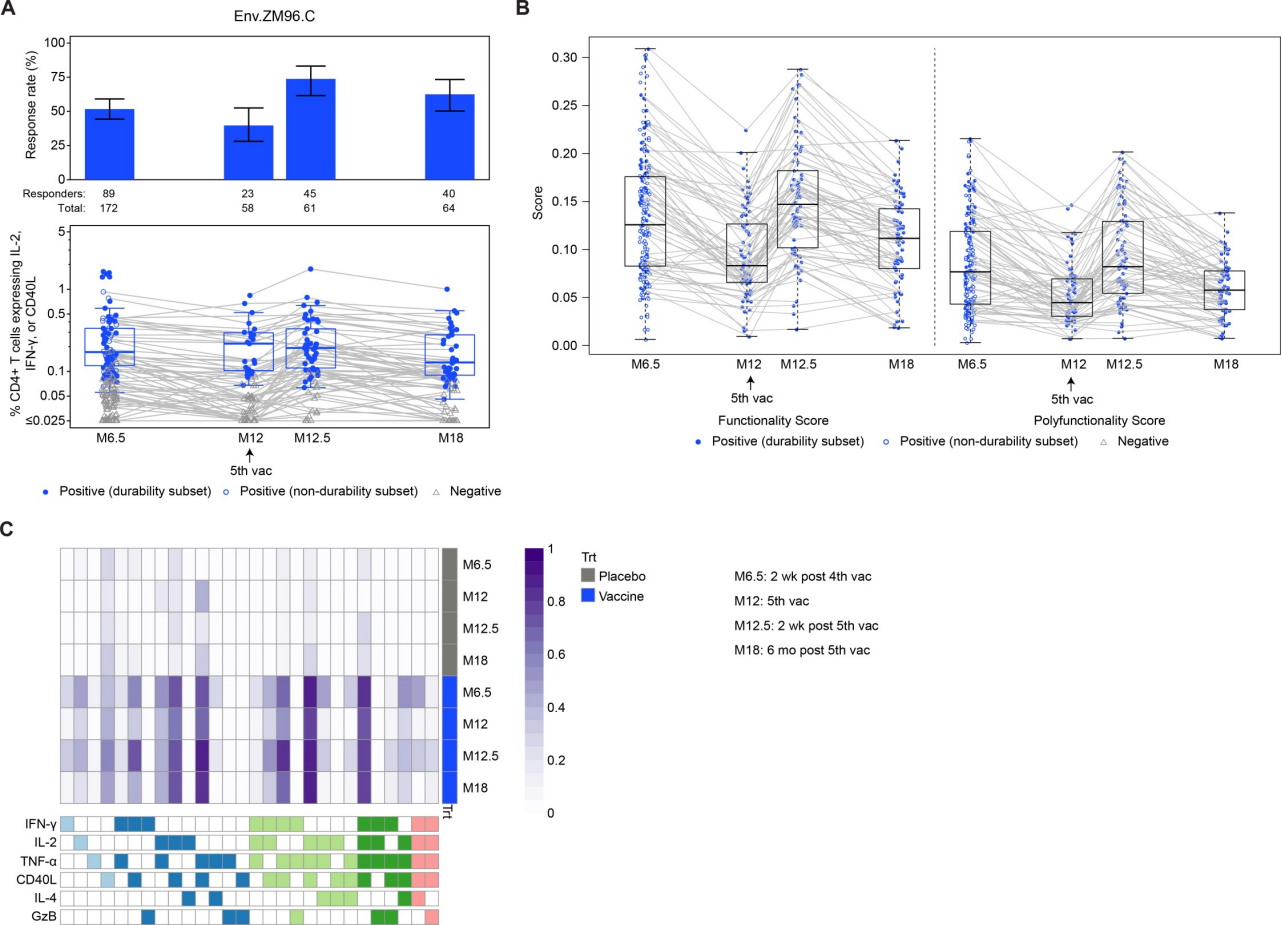
Summary of response rates and magnitudes, functionality and polyfunctionality scores, and heatmap of average COMPASS posterior probabilities of CD4+ T-cell responses to vaccine-matched Env ZM96.C among per-protocol vaccine recipients of HVTN 100. In (A), bar charts show response rates with 2-sided 95% CIs, and boxplots show response magnitude as the percent expression of IFN-γ, IL-2, or CD40L by CD4+ T cells to Env ZM96.C and are based on positive responders, shown as colored circles (solid circles denote durability subset participants, open circles denote non-durability subset participants); negative responders are shown as grey triangles. Boxplots in (B) show functionality and polyfunctionality scores of CD4+ T-cell subsets recognizing Env ZM96.C; solid circles denote durability subset participants, and open circles denote non-durability subset participants. In the boxplots in (A) and (B), grey lines connect the response magnitude of each per-protocol vaccine recipient over time. In (C), each cell of the heatmap shows the average probability, modeled by COMPASS, that a given cell subset (indicated by bottom panel column) has an antigen-specific response at the corresponding timepoint (row), where the probability is color-coded from white (0) to purple (1). The columns in the bottom panel correspond to cellular subsets, color-coded in blue, green, and pink by the cytokines they express. M[number], month [number]; Trt, treatment; vac, vaccination.

As shown in Fig 6A, among per-protocol vaccine recipients, response rates for the vector-matched Env ZM96.C-specific CD4+ T cells expressing IFN-γ, IL-2, or CD40L declined from 60.4% (95% CI = 46.9%–72.4%) at month 6.5 to 41.5% (95% CI = 29.3%–54.9%) at month 12 (*P* = 0.021), but increased after the booster to 73.2% (95% CI = 60.4%–83.0%) at month 12.5 (*P* = 0.14 compared to month 6.5), with a significantly higher response rate of 63.6% at month 18 than 41.8% at month 12 (difference = 21.8%, 95% CI = 5.1%–38.5%, *P* = 0.007). Similar kinetics were seen for the IFN-γ, IL-2, and CD40L CD4+ T-cell responses to the TV1.C and 1086.C peptide pools matched to the gp120 boost immunogens (S3 and S4 Figs). No significant differences were seen in the response magnitude among positive responders to any of the vaccine-matched peptide pools between month 6.5 and 12.5 or between month 12 and 18 (all *P >* 0.43). Higher magnitudes overall (not restricting to positive responders) were seen at month 18 than at month 12 for 1086.C (*P* = 0.030) and for ZM96.C (*P* = 0.009), with some evidence of higher overall magnitude for TV1c8.2.C also (*P* = 0.052); these differences in overall magnitude are likely driven by the difference in response rates. There were no positive responses among placebo recipients at any timepoint. Functionality scores for CD4+ T-cell responses to Env ZM96.C were significantly higher at month 18 than at month 12 (*P* = 0.003, polyfunctionality score *P* = 0.052); however, there was no difference in the functionality and polyfunctionality scores between months 6.5 and 12.5 (*P* = 0.82 and *P* = 0.64, respectively) (Fig 6B). In particular, the COMPASS posterior probabilities for 4- and 5-function CD4+ T-cell responses to Env ZM96.C were similar at months 6.5 and 12.5 as well as at months 12 and 18. However, there appeared to be waning in the 5-function responses at both months 12 and 18, but less in the 4-function responses (pink and dark green 5-function and 4-function subsets in Fig 6C, respectively), compared to the peak timepoints at months 6.5 and 12.5.

The patterns in functionality and polyfunctionality scores that were observed for Env ZM96.C-specific CD4+ T cells were similar for the other vaccine-matched gp120 antigens, 1086.C (S3 Fig) and TV1c8.2.C (S4 Fig).

**Fig 7:**
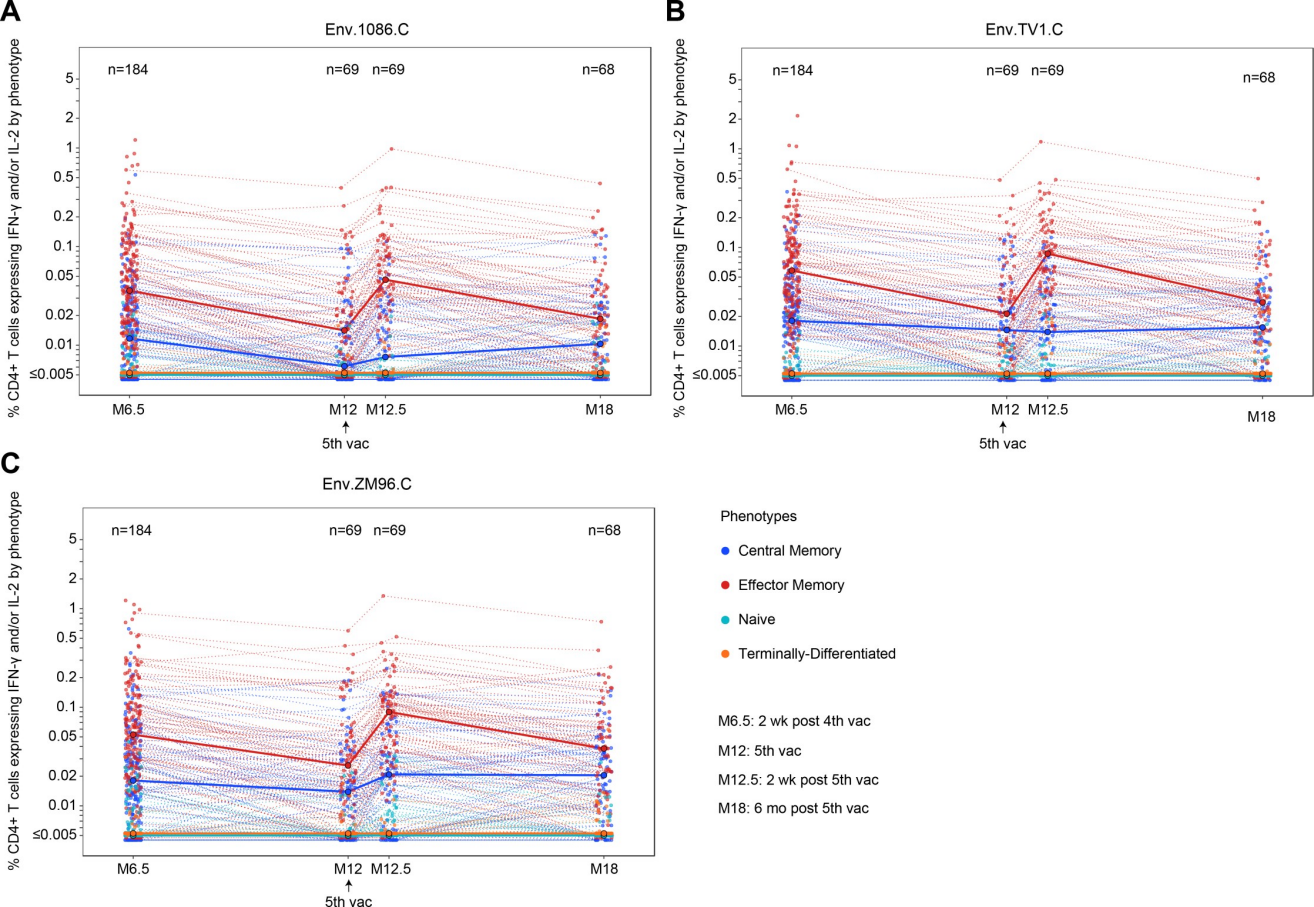
Memory sub-populations of 1086.C gp120, TV1.C gp120, and ZM96.C antigen-specific CD4+ T cells amongst vaccine recipients in HVTN 100. Memory sub-populations of 1086.C (A), TV1.C (B), and ZM96.C (C) antigen-specific CD4+ T cells. Frequencies of central memory (dark blue symbols, CD45RA^−^CCR7^+^), effector memory (red symbols, CD45RA^−^CCR7^−^), naïve (teal symbols, CD45RA^+^CCR7^+^), and terminally differentiated (orange symbols, CD45RA^+^CCR7^−^) CD4+ T cells expressing IFN-γ or IL-2 out of total CD4+ T cells are shown 2 weeks after the fourth vaccination (month 6.5), 6 months after the fourth vaccination (month 12), 2 weeks after the fifth vaccination (month 12.5), and 6 months after the fifth vaccination (month 18). Black circles represent median antigen-specific sub-populations at each timepoint. Dashed lines connect the frequencies of each memory sub-population for each per-protocol vaccine recipient over time. M[number], month [number]; vac, vaccination.

We evaluated the frequencies of sub-populations of vaccine-antigen-specific memory CD4+ T cells in the vaccine group (Fig 7) and in the placebo group (S5 Fig). In the vaccine group, there were changes over the course of the trial in the frequencies of Env 1086.C-specific effector memory CD4+ T cells (expressing IFN-γ or IL-2) as a proportion of total CD4+ T cells, with significant increases observed after the month 12 booster (*P* < 0.001). The frequencies of Env 1086.C-specific effector memory CD4+ T cells contracted significantly from month 6.5 to month 12 (*P* < 0.001) and from month 12.5 to month 18 (*P* < 0.001) but increased significantly from month 12 to 12.5 (*P* < 0.001), with no significant differences seen between months 6.5 and 12.5 (*P* = 0.44) or between months 12 and 18 (*P* = 0.10). Similar patterns were seen in the effector memory phenotypes of the TV1.C- and ZM96.C-specific CD4+ T cells expressing IFN-γ or IL-2 (Fig 7B and 7C). In contrast, the frequencies of Env 1086.C-, TV1.C-, and ZM96.C-specific central memory CD4+ T cells (expressing IFN-γ or IL-2) remained relatively stable throughout, with a slight, but statistically significant, increase for Env ZM96.C-specific responses following the month 12 booster (month 12.5 versus month 12, *P* = 0.002; Fig 7C). As expected, naïve (CD45RA^+^CCR7^+^) and terminally differentiated (CD45RA^+^CCR7^−^) Env 1086.C-, TV1.c-, and ZM96.C-specific CD4+ T cells were low and constant over time (Fig 7A–7C).

### nAb responses

**Fig 8:**
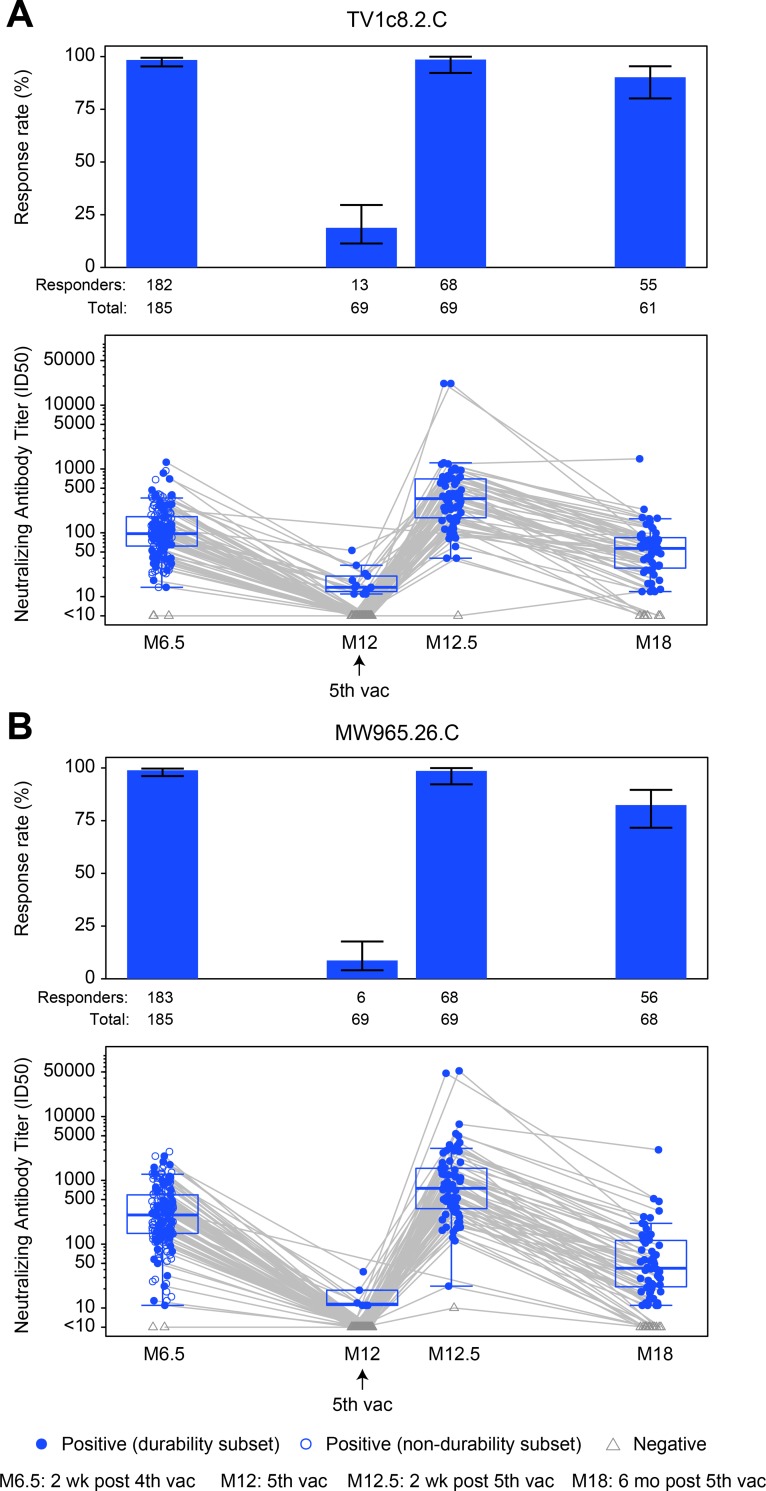
Summary of response rates and magnitudes to vaccine-matched TV1c8.2.C and tier 1A MW965.26.C among per-protocol vaccine recipients of HVTN 100. Responses to (A) TV1c8.2.C and (B) MW965.26.C. Bar charts show response rates with 2-sided 95% CIs. Boxplots show response magnitude as the ID50 neutralizing antibody titer and are based on positive responders, shown as solid blue circles; negative responders are shown as grey triangles. Grey lines connect the response magnitude of each per-protocol vaccine recipient over time. M[number], month [number]; vac, vaccination.

nAbs were assessed against vaccine-matched TV1c8.2.C and heterologous MW965.26.C HIV-1 Env-pseudotyped viruses; both Envs are subtype C and exhibit a tier 1A neutralization phenotype (Fig 8). As seen in Fig 8A and S4 Table, among per-protocol vaccine recipients, the response rates of nAbs to TV1c8.2.C were similarly high at months 12.5 and 6.5: 98.6% versus 97.1% (difference = 1.4%, 95% CI = −4.9% to 7.8%, *P* = 1.00). Durability response rate was significantly higher at month 18 than at month 12: 90.0% versus 16.7% (difference = 73.3%, 95% CI = 60.5%–86.2%, *P* < 0.001). Similarly, there was no difference in the response rate of nAbs to MW965.26.C at months 12.5 and 6.5 (98.6% versus 98.6%, difference = 0.0%, 95% CI = −5.5% to 5.5%, *P* = 1.00), but the response rate were higher at month 18 than at month 12 (83.6% versus 9.0%, difference = 74.6%, 95% CI = 62.7%–86.5%, *P* < 0.001) (Fig 8B). Higher response magnitudes overall and among positive responders were seen at month 12.5 compared to month 6.5 for TV1c8.2.C (both *P* < 0.001), as well as higher response magnitudes overall and among positive responders at month 18 versus month 12 (*P* < 0.001 and *P* = 0.006, respectively). Similarly, higher response magnitudes overall and among positive responders were seen at month 12.5 compared to month 6.5 for MW965.26.C (both *P* < 0.001), as well as higher response magnitudes overall and among positive responders at month 18 versus month 12 (*P* < 0.001 and *P* = 0.03, respectively).

Tier 2 nAbs were assessed at month 12.5 using the 2 vaccine strains 96ZM651.C and Ce1086_B2.C plus a global panel [24] and a clade C panel [25] of reference strains; no activity was seen against any of these 26 viruses. Because no nAb responses against tier 2 viruses were detected at month 12.5, they were not assessed at further timepoints. Assays also were performed at month 6.5 with viruses that detect early precursors of certain VRC01 class broadly neutralizing antibodies [26]; the results were all negative.

## Discussion

Our study, HVTN 100, demonstrates that the durability of immune responses to an HIV vaccine regimen can be extended by the administration of a fifth vaccination booster given at month 12. Furthermore, the study also shows that the vaccines were well tolerated and have an acceptable safety profile.

An ongoing challenge in the HIV vaccine field remains inducing immune responses that are durable. Response durability could plausibly be influenced by product-related factors such as vaccine design, dose, administration route, schedule, and adjuvants. Regimens that include more immunogenic adjuvants and repeated vaccination may help to extend and enhance levels of protective immunity for longer periods of time after vaccination [27]. Follow-up booster vaccinations have the potential to augment protective immunity; the maturation of the immune response can lead to increased affinity for antigen. The RV305 study, in which vaccine boosters were given 6–8 years after the primary regimen, observed expansion in a sub-pool of memory B cells whose germline precursors and affinity-matured B cell clonal lineage members neutralized a tier 2 HIV isolate [28,29]. Additionally, durability of the immune response could be influenced by host-related factors such as antibody titers resulting from vaccination, host genetics (e.g., HLA type) [30], health history, and microbiome. For some biomarkers and for some vaccines, including those administered in the RV144 trial, 2-week post-vaccination responses predict durable responses 6 months later [31]. Our study of a heterologous prime–boost regimen demonstrates immune responses that vary considerably amongst individuals, suggesting that host factors contribute substantially to immune response durability.

The HVTN 100 vaccine regimen was similar to the RV144 regimen, but there were regional adaptations to the immunogens and modifications to the regimen in an effort to extend durability. Immune responses 2 weeks after the fourth vaccination were at least comparable to those observed in the RV144 trial. A fifth vaccination was given at month 12 in HVTN 100 to extend the levels of vaccine-induced immune responses. With the fifth vaccination, we observed that, in general, immune responses could be significantly boosted, and that thereafter the response rates and magnitudes decayed more slowly than after the fourth vaccination. We observed significant enhancement of IgG and IgG3 bAb, ADCP, tier 1A nAb, and CD4+ T-cell responses 2 weeks after the booster, with the expected waning of immunity over the subsequent 6-month time period. Notably, some antibody responses were persistent (e.g., IgG responses to the 1086.C vaccine-matched antigen and ADCP responses to 1086.C gp140), indicating that potentially long-lived memory B cells were stimulated by vaccination.

Our results demonstrate that the month 12 booster of the subtype C pox-protein vaccine regimen can restore both tier 1A neutralizing and non-neutralizing antibody responses to peak levels after 2 weeks, and that these levels are sustained reasonably well for 6 months after the booster. This regimen, like that in the RV144 trial, has been able to neutralize only tier 1A HIV isolates, indicating that inducing a broader tier 2 neutralization requires an alternative approach.

Decreased HIV acquisition risk has previously been associated in RV144 vaccine recipients with polyfunctional CD4+ T cells producing multiple cytokines (tumor necrosis factor alpha, interferon-gamma, interleukin-4, interleukin-2, and CD40L) in response to HIV-1 Env antigen [18]. Although the booster served to restore T-cell functionality and polyfunctionality initially, these also waned over time.

Our study focused on the immunogenicity and durability of immune responses induced by this subtype C pox-protein vaccine regimen adapted to the sub-Saharan population, and was not designed to answer clinical questions around vaccine efficacy. The HVTN 702 trial, which has completed enrollment of 5,407 participants and is currently ongoing in South Africa, is designed to provide evidence on the efficacy of this vaccine regimen from 0 to 36 months after enrollment. The durability data from HVTN 100 presented here support the vaccine schedule utilized in HVTN 702, which includes a booster at month 12 along with a subsequent booster at month 18 in order to extend even further the total immune response coverage over time. Decay of measurable antibody and cellular immune responses may or may not signify waning of protection; likewise, restoration of immune responses may or may not improve protection. However, it is possible that even if immune responses dip to low levels, upon exposure a rapid recall response that prevents the establishment of infection could occur.

More than 10,000 human volunteers have received other ALVAC-HIV recombinant canarypox vector vaccine constructs, mostly vCP205, vCP1452, and vCP1521 [1,32]. However, this paper provides to our knowledge the first description of safety data for vCP2438, the construct of ALVAC-HIV developed against HIV subtype C. Similarly, although recombinant monomeric gp120 subunit vaccine formulations have been tested in many clinical trials, ours is the first safety profile description to our knowledge for bivalent subtype C gp120/MF59. MF59 is a registered adjuvant with an established safety record. Our trial results demonstrate a satisfactory clinical safety profile and support further administration of ALVAC-HIV (vCP2438) and bivalent subtype C gp120/MF59 vaccine to humans, as in the HVTN 702 efficacy trial currently underway.

A limitation of our trial design was that it did not evaluate higher doses of gp120, which may have increased the durability of the immune response. The gp120 dose administered in HVTN 100 was one-third the dose used in the RV144 trial, with the expectation that MF59 would be dose-sparing relative to the alum adjuvant [33]. There are 2 caveats in interpretation of our data. First, this study was conducted in a population of healthy young adults at low risk for HIV acquisition in South Africa, and it is unknown whether findings can be generalized to different populations. Second, this study was not designed to investigate efficacy. Further evaluation of the full breadth of antibody Fc effector functions (e.g., ADCC [8,34] and complement binding [35]) is needed to understand the depth of potentially protective immunity elicited by this vaccine. To inform future vaccine immunogen design, further evaluation is needed of the antibody specificities that comprise the antibody functions and the kinetics of the antibody isotype [8] and subclass profile [5,21] after boosting, and how they relate to antiviral functions.

### Conclusion

Based on the decay kinetics of vaccine-induced immune responses of pox-protein heterologous prime–boost regimens, it seems that additional boosters are required to improve the immune response durability. We demonstrate that a 12-month booster of subtype C pox-protein vaccines boosts bAb, tier 1A nAb, and ADCP responses, which persist in many participants to 18 months after initial vaccination, and prolongs robust CD4+ T-cell, ADCP, and tier 1A nAb responses. Further research is also being conducted with a subset of the HVTN 100 trial participants to evaluate immune responses after a late booster at month 30.

## Supporting information

S1 CONSORT Checklist HVTN 100 CONSORT checklist. (DOC)

S1 Fig Summary of response rates and magnitudes of IgG3 binding antibodies to gp120 and V1V2 antigens among per-protocol vaccine recipients of HVTN 100. Bar charts show response rates with 2-sided 95% CIs. Boxplots show magnitude as log_10_ (MFI-blank) responses to individual antigens and are based on positive responders, shown as solid blue circles; negative responders are shown as grey triangles.
(TIF)

S2 Fig Breadth of IgG3 binding antibody responses to gp120, gp140, and V1V2 antigens among per-protocol vaccine recipients of HVTN 100. Responses to gp120 (A), gp140 (B), and V1V2 (C) antigens. Each point represents the area under the magnitude–breadth curve for an individual vaccine recipient, calculated as the average of the log_10_ (MFI-blank) over the panel of antigens, where antigens are listed in the footnote below each plot.
(TIF)

S3 Fig Summary of response rates and magnitudes, functionality and polyfunctionality scores, and heatmap of COMPASS posterior probabilities of CD4+ T-cell responses to vaccine-matched Env 1086.C among per-protocol vaccine recipients of HVTN 100. In (A), bar charts show response rates with 2-sided 95% CIs, and boxplots show magnitude as the percent expression of IFN-γ, IL-2, or CD40L by CD4+ T cells to Env 1086.C and are based on positive responders, shown as colored circles; negative responders are shown as grey triangles. Boxplots in (B) show functionality and polyfunctionality scores of CD4+ T-cell subsets recognizing Env 1086.C. In (C), columns correspond to cellular subsets modeled by COMPASS, color-coded by the cytokines they express. Each cell of the heatmap shows the probability that a given cell subset (column) has an antigen-specific response in the corresponding participant (column), where the probability is color-coded from white (0) to purple (1).
(TIF)

S4 Fig Summary of response rates and magnitudes, functionality and polyfunctionality scores, and heatmap of COMPASS posterior probabilities of CD4+ T-cell responses to vaccine-matched Env TV1c8.2.C among per-protocol vaccine recipients of HVTN 100. In (A), bar charts show response rates with 2-sided 95% CIs, and boxplots show magnitude as the percent expression of IFN-γ, IL-2, or CD40L by CD4+ T cells to TV1c8.2.C and are based on positive responders, shown as colored circles; negative responders are shown as grey triangles. Boxplots in (B) show functionality and polyfunctionality scores of CD4+ T-cell subsets recognizing Env TV1c8.2.C. In (C), columns correspond to cellular subsets modeled by COMPASS, color-coded by the cytokines they express. Each cell of the heatmap shows the probability that a given cell subset (column) has an antigen-specific response in the corresponding participant (column), where the probability is color-coded from white (0) to purple (1).
(TIF)

S5 Fig Memory sub-populations of 1086.C gp120, TV1.C gp120, and ZM96.C antigen-specific CD4+ T cells amongst placebo recipients of HVTN 100. Memory sub-populations of 1086.C (A), TV1.C (B), and ZM96.C (C) antigen-specific CD4+ T cells. Frequencies of central memory (dark blue symbols, CD45RA^−^CCR7^+^), effector memory (red symbols, CD45RA^−^CCR7^−^), naïve (teal symbols, CD45RA^+^CCR7^+^), and terminally differentiated (orange symbols, CD45RA^+^CCR7^−^) CD4+ T cells expressing IFN-γ or IL-2 out of total CD4+ T cells are shown 2 weeks after the fourth vaccination (month 6.5), 6 months after the fourth vaccination (month 12), 2 weeks after the fifth vaccination (month 12.5), and 6 months after the fifth vaccination (month 18). Black circles represent median antigen-specific sub-populations at each timepoint.
(TIF)

S1 Table Participant baseline characteristics of the HVTN 100 intention-to-treat cohort (*n* = 252), the per-protocol cohort (*n* = 222), and the durability subset (*n* = 75). (DOCX)

S2 Table Details of the binding antibody multiplex assay, intracellular cytokine staining, and neutralizing antibody antigens used in laboratory assays, including HIV-1 viral strain information. (DOCX)

S3 Table Response rates (95% CIs) and geometric mean (GM) magnitudes (95% CIs) overall and among positive responders of primary humoral and cellular responses at peak (months 6.5 and 12.5) and durability (months 12 and 18) timepoints. (DOCX)

S4 Table Response rates (95% CIs) and geometric mean (GM) magnitudes (95% CIs) overall and among positive responders of secondary and exploratory humoral and cellular responses at peak (months 6.5 and 12.5) and durability (months 12 and 18) timepoints. Effector and central memory sub-populations are exploratory.
(DOCX)

## Abbreviations

ADCP: antibody-dependent cellular phagocytosis
AE: adverse event
AUC: area under the curve
bAb: binding antibody
gp120: glycoprotein 120
HVTN: HIV Vaccine Trials Network
nAb: neutralizing antibody
