# Supplementary figures and images for: Safety and immune responses after a 12-month booster in healthy HIV-uninfected adults in HVTN 100 in South Africa: A randomized double-blind placebo-controlled trial of ALVAC-HIV (vCP2438) and bivalent subtype C gp120/MF59 vaccines
Source: PLoS Med. 2020 Feb 24;17(2):e1003038. doi: 10.1371/journal.pmed.1003038 (PMC7039414; doi:10.1371/journal.pmed.1003038)

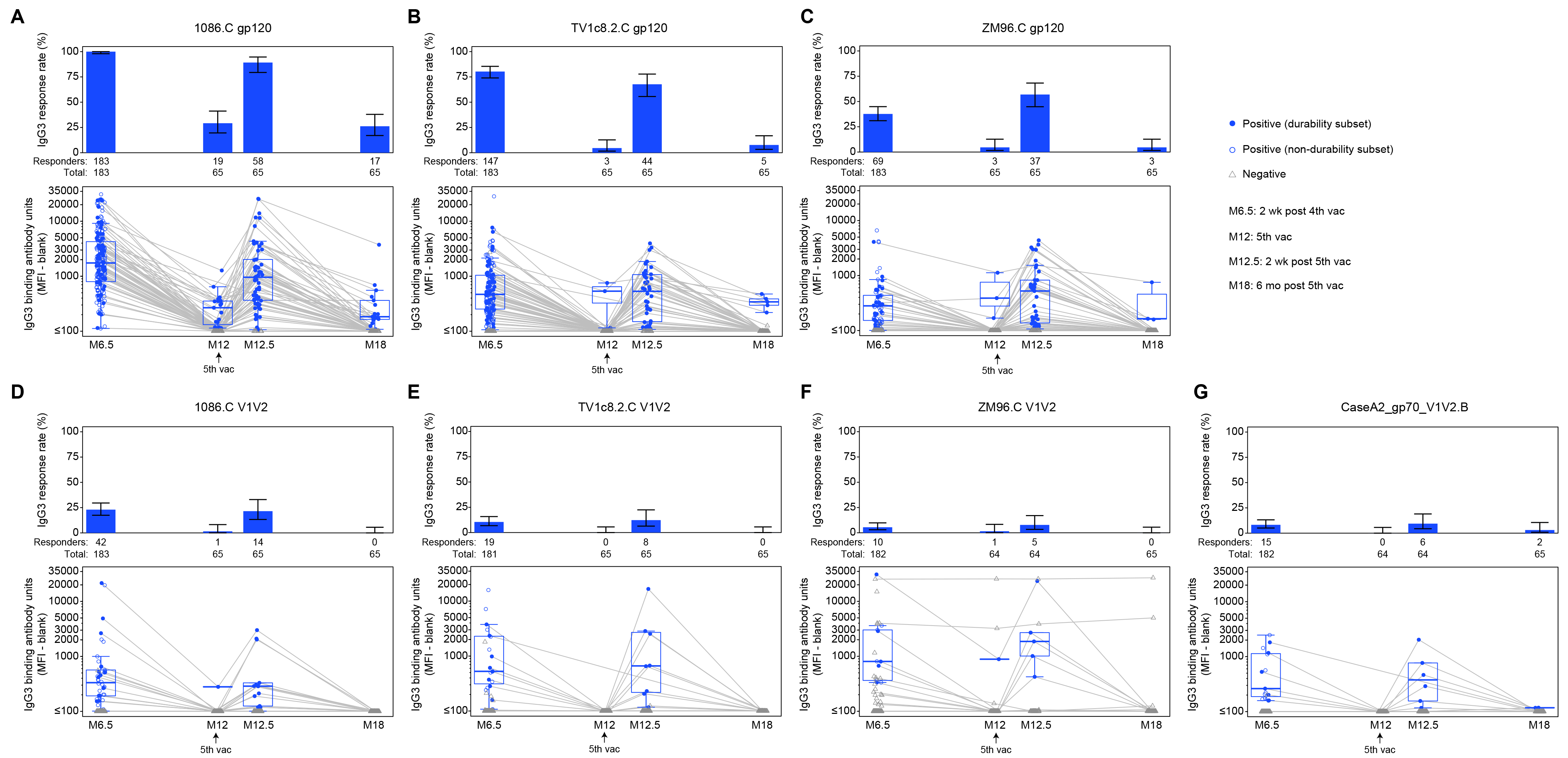

Supplement: S1 Fig — Bar charts show response rates with 2-sided 95% CIs. Boxplots show magnitude as log10 (MFI-blank) responses to individual antigens and are based on positive responders, shown as solid blue circles; negative responders are shown as grey triangles. (TIF) [file pmed.1003038.s002.tif]

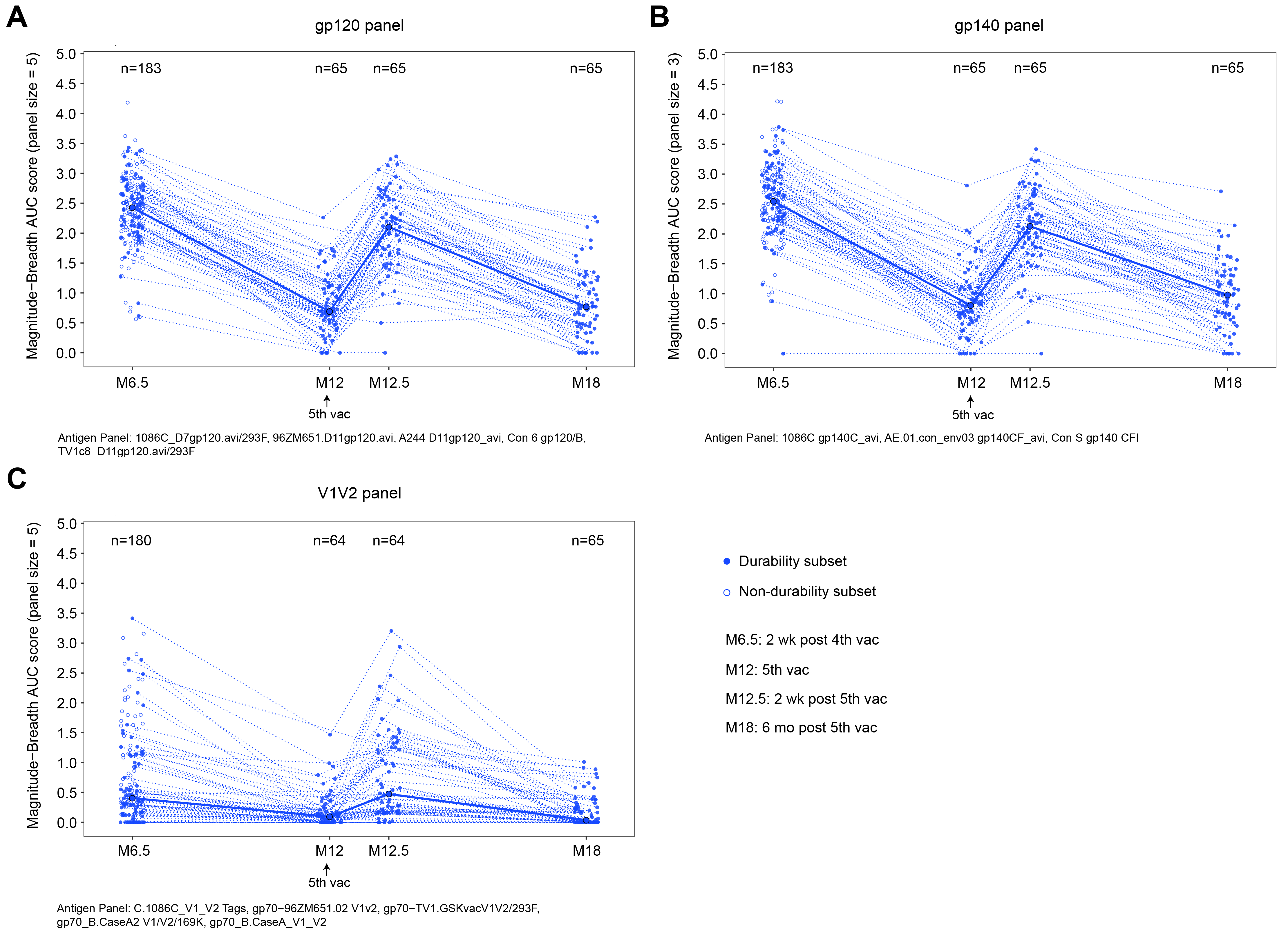

Supplement: S2 Fig — Responses to gp120 (A), gp140 (B), and V1V2 (C) antigens. Each point represents the area under the magnitude–breadth curve for an individual vaccine recipient, calculated as the average of the log10 (MFI-blank) over the panel of antigens, where antigens are listed in the footnote below each plot. (TIF) [file pmed.1003038.s003.tif]

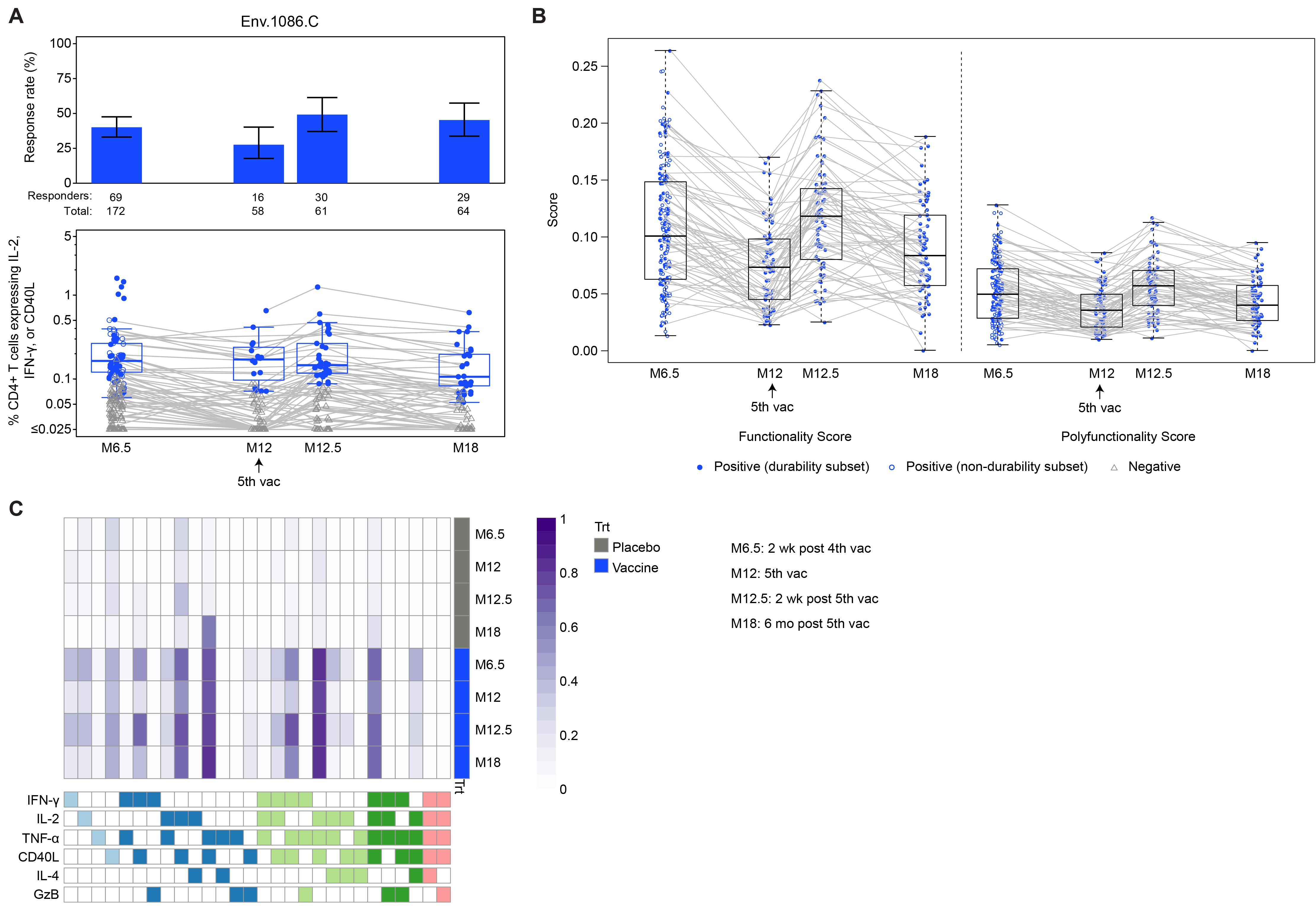

Supplement: S3 Fig — In (A), bar charts show response rates with 2-sided 95% CIs, and boxplots show magnitude as the percent expression of IFN-γ, IL-2, or CD40L by CD4+ T cells to Env 1086.C and are based on positive responders, shown as colored circles; negative responders are shown as grey triangles. Boxplots in (B) show functionality and polyfunctionality scores of CD4+ T-cell subsets recognizing Env 1086.C. In (C), columns correspond to cellular subsets modeled by COMPASS, color-coded by the cytokines they express. Each cell of the heatmap shows the probability that a given cell subset (column) has an antigen-specific response in the corresponding participant (column), where the probability is color-coded from white (0) to purple (1). (TIF) [file pmed.1003038.s004.tif]

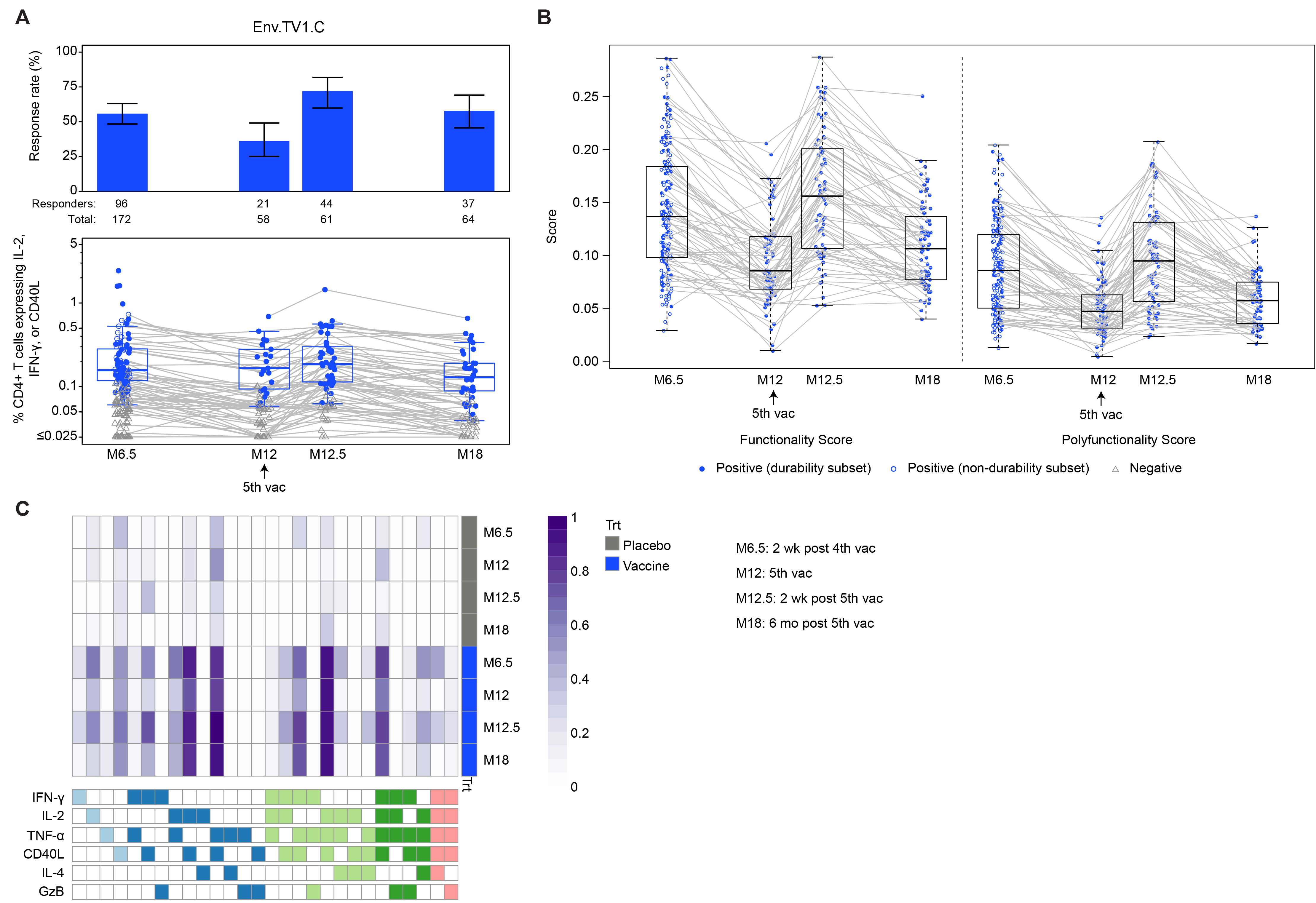

Supplement: S4 Fig — In (A), bar charts show response rates with 2-sided 95% CIs, and boxplots show magnitude as the percent expression of IFN-γ, IL-2, or CD40L by CD4+ T cells to TV1c8.2.C and are based on positive responders, shown as colored circles; negative responders are shown as grey triangles. Boxplots in (B) show functionality and polyfunctionality scores of CD4+ T-cell subsets recognizing Env TV1c8.2.C. In (C), columns correspond to cellular subsets modeled by COMPASS, color-coded by the cytokines they express. Each cell of the heatmap shows the probability that a given cell subset (column) has an antigen-specific response in the corresponding participant (column), where the probability is color-coded from white (0) to purple (1). (TIF) [file pmed.1003038.s005.tif]

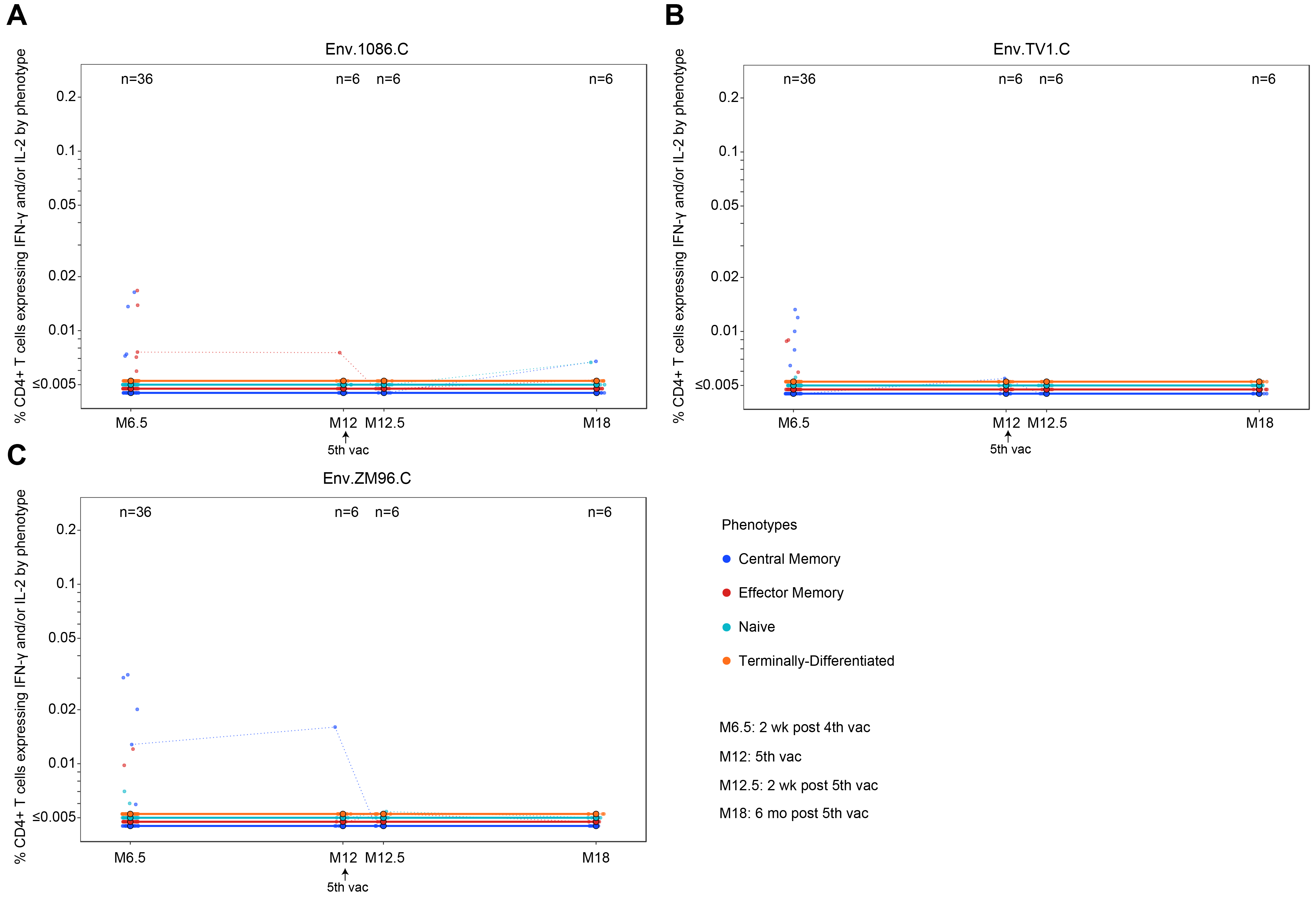

Supplement: S5 Fig — Memory sub-populations of 1086.C (A), TV1.C (B), and ZM96.C (C) antigen-specific CD4+ T cells. Frequencies of central memory (dark blue symbols, CD45RA−CCR7+), effector memory (red symbols, CD45RA−CCR7−), naïve (teal symbols, CD45RA+CCR7+), and terminally differentiated (orange symbols, CD45RA+CCR7−) CD4+ T cells expressing IFN-γ or IL-2 out of total CD4+ T cells are shown 2 weeks after the fourth vaccination (month 6.5), 6 months after the fourth vaccination (month 12), 2 weeks after the fifth vaccination (month 12.5), and 6 months after the fifth vaccination (month 18). Black circles represent median antigen-specific sub-populations at each timepoint. (TIF) [file pmed.1003038.s006.tif]
