# Supplementary material for: Safety and immune responses after a 12-month booster in healthy HIV-uninfected adults in HVTN 100 in South Africa: A randomized double-blind placebo-controlled trial of ALVAC-HIV (vCP2438) and bivalent subtype C gp120/MF59 vaccines
Source: PLoS Med. 2020 Feb 24;17(2):e1003038. doi: 10.1371/journal.pmed.1003038 (PMC7039414; doi:10.1371/journal.pmed.1003038)
Supplement: S1 Table — (DOCX) [file pmed.1003038.s007.docx]

**S1 Table. Participant baseline characteristics of the HVTN 100 intention-to-treat cohort (n=252), the per-protocol* cohort (n=222), and the durability subset (n=75).**

|  | **Intention-to-Treat** | | | **Per-Protocol** | | | **Durability Subset** | | |
| --- | --- | --- | --- | --- | --- | --- | --- | --- | --- |
|  | **Vaccine-recipients**  N (% of vaccine-recipients) | **Placebo-recipients**  N (% of placebo-recipients) | **Total**  N (% of total participants) | **Vaccine-recipients**  N (% of vaccine-recipients) | **Placebo-recipients**  N (% of placebo-recipients) | **Total**  N (% of total participants) | **Vaccine-recipients**  N (% of vaccine-recipients) | **Placebo-recipients**  N (% of placebo-recipients) | **Total**  N (% of total participants) |
| **Number (%)** | 210 (100%) | 42 (100%) | 252 (100%) | 185 (100%) | 37 (100%) | 222 (100%) | 70 (100%) | 5 (100%) | 75 (100%) |
| **Median age in years (IQR)** | 23.0 (21.0-27.0) | 23.0 (21.0-26.0) | 23.0 (21.0-27.0) | 23.0 (21.0-27.0) | 23.0 (21.0-26.0) | 23.0 (21.0-27.0) | 24.0 (21.0-28.0) | 26.0 (24.0-26.0) | 24.0 (21.0-28.0) |
| **Sex¶** |  |  |  |  |  |  |  |  |  |
| Female | 88 (41.9%) | 21 (50.0%) | 109 (43.3%) | 73 (39.5%) | 18 (48.6%) | 91 (41%) | 28 (40.0%) | 2 (40.0%) | 30 (40.0%) |
| Male | 122 (58.1%) | 21 (50.0%) | 143 (56.7%) | 112 (60.5%) | 19 (51.4%) | 131 (59%) | 42 (60.0%) | 3 (60.0%) | 45 (60.0 %) |
| **Body-mass index (kg/m^2^)** |  |  |  |  |  |  |  |  |  |
| <18.5 | 24 (11.4%) | 3 (7.1%) | 27 (10.7%) | 20 (10.8%) | 2 (5.4%) | 22 (9.9%) | 7 (10.0%) | 0 (0%) | 7 (9.3%) |
| 18.5-24.99 | 115 (54.8%) | 20 (47.6%) | 135 (53.6%) | 104 (56.2%) | 19 (51.4%) | 123 (55.4%) | 40 (57.1%) | 2 (40.0%) | 42 (56.0%) |
| 25-29.99 | 40 (19.0%) | 12 (28.6%) | 52 (20.6%) | 36 (19.5%) | 10 (27.0%) | 46 (20.7%) | 13 (18.6%) | 3 (60.0%) | 16 (21.3%) |
| >=30 | 31 (14.8%) | 7 (16.7%) | 38 (15.1%) | 25 (13.5%) | 6 (16.2%) | 31 (14.0%) | 10 (14.3%) | 0 (0%) | 10 (13.3%) |
| **Highest level of education** |  |  |  |  |  |  |  |  |  |
| Primary School | 7 (3.3%) | 0 (0%) | 7 (2.8%) | 6 (3.2%) | 0 (0%) | 6 (2.7%) | 2 (2.9%) | 0 (0%) | 2 (2.7%) |
| High School | 157 (74.8%) | 33 (78.6%) | 190 (75.4%) | 136 (73.5%) | 28 (75.7%) | 164 (73.9%) | 53 (75.7%) | 2 (40.0%) | 55 (73.3%) |
| Tertiary/College/  University | 46 (21.9%) | 9 (21.4%) | 55 (21.8%) | 43 (23.2%) | 9 (24.3%) | 52 (23.4%) | 15 (21.4%) | 3 (60.0%) | 18 (24.0%) |

Data are listed as number of participants (%). Percentages may not always add up to 100% due to rounding.

¶Sex options included trans and self-identify. One participant self-identified as homosexual male; all other participants reported male or female.

*Per-protocol cohort includes participants who received the first four scheduled vaccinations and did not have HIV infection at month 6.5; two participants were infected with HIV before month 6.5.
