# Supplementary material for: Safety and immune responses after a 12-month booster in healthy HIV-uninfected adults in HVTN 100 in South Africa: A randomized double-blind placebo-controlled trial of ALVAC-HIV (vCP2438) and bivalent subtype C gp120/MF59 vaccines
Source: PLoS Med. 2020 Feb 24;17(2):e1003038. doi: 10.1371/journal.pmed.1003038 (PMC7039414; doi:10.1371/journal.pmed.1003038)
Supplement: S2 Table — (DOCX) [file pmed.1003038.s008.docx]

**S2 Table. Details of the BAMA, ICS, and nAb antigens used in laboratory assays, including HIV-1 viral strain information.**

| **Figure** | **Assay** | **Antigen class** | **Label used in plot** | **Antigen** | **Viral strain information: Subtype.Country.Year.Stage*** |
| --- | --- | --- | --- | --- | --- |
| Fig3 (IgG) and S1 Fig (IgG3) | BAMA | gp120 | 1086.C gp120 | 1086C_D7gp120.avi/293F | C.MW.04.1-2 |
|  |  |  | TV1c8.2.C gp120 | TV1c8_D11gp120.avi/293F | C.ZA.98.6 |
|  |  |  | ZM96.C gp120 | 96ZM651.D11gp120.avi | C.ZM.96.6 |
|  |  | V1V2 | 1086.C V1V2 | C.1086C_V1_V2 Tags | C.MW.04.1-2 |
|  |  |  | TV1c8.2.C V1V2 | gp70-TV1.GSKvacV1V2/293F | C.ZA.98.6 |
|  |  |  | ZM96.C V1V2 | gp70-96ZM651.02 V1v2 | C.ZM.96.6 |
|  |  |  | CaseA2_gp70_V1V2.B | gp70_B.CaseA_V1_V2 | B.US.88.6 |
| Fig4  (IgG breadth panel) | BAMA (IgG breadth panel) | gp120 | gp120 panel | 254008_D11gp120.avi/293F | CRF01_AE.TH.09.2 |
|  |  |  |  | 51802_D11gp120.avi/293F | A1.KE.09.1 |
|  |  |  |  | A244 D11gp120_avi | CRF01_AE.TH.90.6 |
|  |  |  |  | B.6240_D11gp120/293F | B.US.95.2 |
|  |  |  |  | BJOX002_D11gp120.avi/293F | CRF07_BC.CN.07.1-2 |
|  |  |  |  | BORI_D11gp120.avi/293F | B.US.90.2 |
|  |  |  |  | CNE20_D11gp120.avi/293F | CRF07_BC.CN.07.6 |
|  |  |  |  | TT31P.2792_D11gp120.avi/293F | B.TT.98.2 |
|  |  | gp140 | gp140 panel | 1086C gp140C_avi | C.MW.04.1-2 |
|  |  |  |  | 9004S.gp140C.avi | A1.UG.07.4 |
|  |  |  |  | AE.01.con_env03 gp140CF_avi | CRF01_AE.xx.xx |
|  |  |  |  | BF1266_gp140C.avi/293F | C.MW.02.1-2 |
|  |  |  |  | C.CH505TF_gp140/293F | C.MW.08.4 |
|  |  |  |  | RHPA4259_C7.gp140C.avi | B.US.00.5 |
|  |  |  |  | SC42261_gp140.avi/293F | B.TT.95.4 |
|  |  |  |  | WITO4160.gp140C.avi | B.US.00.2 |
|  |  | V1V2 | V1V2 panel | gp70−001428.2.42 V1V2 | C.IN.00.4 |
|  |  |  |  | gp70−191084_B7 V1V2 | A1.UG.07.4 |
|  |  |  |  | gp70−62357.14 V1V2 | B.US.96.2 |
|  |  |  |  | gp70−700010058 V1V2 | B.US.06.3 |
|  |  |  |  | gp70−7060101641 V1V2 | C.ZA.07.3 |
|  |  |  |  | gp70−96ZM651.02 V1v2 | C.ZM.96.6 |
|  |  |  |  | gp70−BF1266_431a_V1V2 | C.MW.02.1-2 |
|  |  |  |  | gp70−BJOX002000.03.2 V1V2 | CRF07_BC.CN.07.1-2 |
|  |  |  |  | gp70−C2101.c01_V1V2 | CRF01_AE.TH.99.5-6 |
|  |  |  |  | gp70−CAP210.2.00.E8 V1V2 | C.ZA.05.4 |
|  |  |  |  | gp70−CM244.ec1 V1V2 | CRF01_AE.TH.90.6 |
|  |  |  |  | gp70−RHPA4259.7 V1V2 | B.US.00.5 |
|  |  |  |  | gp70−TT31P.2F10.2792 V1V2 | B.TT.98.2 |
|  |  |  |  | gp70−TV1.GSKvacV1V2/293F | C.ZA.98.6 |
|  |  |  |  | gp70_B.CaseA_V1_V2 | B.US.88.6 |
|  |  |  |  | gp70_C.1086C V1/V2/293F | C.MW.04.1-2 |
| S2 Fig  (IgG3 breadth panel) | BAMA (IgG3 breadth panel) | gp120 | gp120 panel | 1086C_D7gp120.avi/293F | C.MW.04.1-2 |
|  |  |  |  | 96ZM651.D11gp120.avi | C.ZM.96.6 |
|  |  |  |  | A244 D11gp120_avi | CRF01_AE.TH.90.6 |
|  |  |  |  | Con 6 gp120/B | [Group M Consensus] |
|  |  |  |  | TV1c8_D11gp120.avi/293F | C.ZA.98.6 |
|  |  | gp140 | gp140 panel | 1086C gp140C_avi | C.MW.04.1-2 |
|  |  |  |  | AE.01.con_env03 gp140CF_avi | CRF01_AE.xx.xx |
|  |  |  |  | Con S gp140 CFI | [Group M Consensus] |
|  |  | V1V2 | V1V2 panel | C.1086C_V1_V2 Tags | C.MW.04.1-2 |
|  |  |  |  | gp70−96ZM651.02 V1v2 | C.ZM.96.6 |
|  |  |  |  | gp70−TV1.GSKvacV1V2/293F | C.ZA.98.6 |
|  |  |  |  | gp70_B.CaseA2 V1/V2/169K | B.US.88.6 |
|  |  |  |  | gp70_B.CaseA_V1_V2 | B.US.88.6 |
| Fig5, S3 Fig, S4 Fig (ICS); Fig 6 and S5 Fig (Phenotyping) | ICS | -  -  - | Env.ZM96.C | ZM96 gp120 | C.ZM.96.6 |
|  |  |  | Env.1086.C | 1086 gp120 | C.MW.04.1-2 |
|  |  |  | Env.TV1.C | TV1 gp120 | C.ZA.98.6 |
| Fig 7 | nAb | EPV** | TV1c8.2.C | TV1c8.2 | C.ZA.98.6 |
|  |  |  | MW965.26.C | MW965.26 | C.MW.93.6 |
| *Subtype is denoted by a capital letter; country of origin is denoted by the 2 digit International Organization for Standardization code; year isolated is denoted by 2 digits; and stage is denoted by “a” (acute, if Fiebig stage is unknown) or “1”, “2”, “3”, “4”, “5”, or “6” (acute or early chronic, where the number or range corresponds to the Fiebig stage or range of stages when known).  **EPV = Env-pseudotyped virus | | | | | |
