# Supplementary material for: Safety and immune responses after a 12-month booster in healthy HIV-uninfected adults in HVTN 100 in South Africa: A randomized double-blind placebo-controlled trial of ALVAC-HIV (vCP2438) and bivalent subtype C gp120/MF59 vaccines
Source: PLoS Med. 2020 Feb 24;17(2):e1003038. doi: 10.1371/journal.pmed.1003038 (PMC7039414; doi:10.1371/journal.pmed.1003038)
Supplement: S3 Table — (DOCX) [file pmed.1003038.s009.docx]

**S3 Table:** **Response rates (95% CIs) and geometric mean (GM) magnitudes (95% CIs) overall and among positive responders of primary humoral and cellular responses at peak (Months 6.5, 12.5) and durability (Months 12, 18) timepoints.**

| **Endpoint** | **Statistic** | **Peak Timepoints** | | **Durability Timepoints** | |
| --- | --- | --- | --- | --- | --- |
|  |  | **Month 6.5**  **Estimate**  **(95% CI)** | **Month 12.5**  **Estimate**  **(95% CI)** | **Month 12 Estimate**  **(95% CI)** | **Month 18 Estimate**  **(95% CI)** |
| **IgG**  **ZM96.C**  **gp120** | **Response rate** | 60/65 = 92.3% (83.2%, 96.7%) | 59/65 = 90.8% (81.3%, 95.7%) | 1/62 = 1.6% (0.3%, 8.6%) | 40/62 = 64.5% (52.1%, 75.3%) |
|  | **GM**  **(overall)** | 25690 (24589, 26840) | 24283 (17328, 34028) | 595 (320, 1107) | 4672 (3152, 6925) |
|  | **GM**  **(among positive responders)** | 25455 (24293, 26672) | 30015 (28637, 31460) | 2944 (-, -) | 7226 (5794, 9011) |
| **IgG**  **1086.C**  **gp120** | **Response rate** | 65/65 =100.0% (94.4%, 100.0%) | 64/65 = 98.5% (91.8%, 99.7%) | 62/62 =100.0% (94.2%, 100.0%) | 61/62 = 98.4% (91.4%, 99.7%) |
|  | **GM**  **(overall)** | 28749 (28206, 29304) | 26288 (19116, 36150) | 8802 (7068, 10963) | 17234 (12174, 24397) |
|  | **GM**  **(among positive responders)** | 28749 (28206, 29304) | 30819 (30075, 31581) | 8802 (7068, 10963) | 20223 (17606, 23228) |
| **IgG**  **TV1c8.2.C**  **gp120** | **Response rate** | 65/65 =100.0% (94.4%, 100.0%) | 64/65 = 98.5% (91.8%, 99.7%) | 56/62 = 90.3% (80.5%, 95.5%) | 61/62 = 98.4% (91.4%, 99.7%) |
|  | **GM**  **(overall)** | 27843 (27228, 28472) | 24917 (17830, 34823) | 556 (332, 931) | 5502 (3687, 8212) |
|  | **GM**  **(among positive responders)** | 27843 (27228, 28472) | 29188 (26093, 32650) | 960 (727, 1267) | 6337 (4748, 8458) |
| **IgG ZM96.C V1V2** | **Response rate** | 29/65 = 44.6% (33.2%, 56.7%) | 44/65 = 67.7% (55.6%, 77.8%) | 4/62 = 6.5% (2.5%, 15.4%) | 13/62 = 21.0% (12.7%, 32.6%) |
|  | **GM (overall)** | 410 (227, 738) | 1310 (715, 2403) | 9.21 (4.48, 18.93) | 22.9 (9.96, 52.64) |
|  | **GM (among positive responders)** | 2018 (1323, 3079) | 4190 (2876, 6104) | 2638 (461, 15086) | 1517 (772, 2983) |
| **IgG 1086.C V1V2** | **Response rate** | 49/65 = 75.4% (63.7%, 84.2%) | 57/65 = 87.7% (77.5%, 93.6%) | 6/62 = 9.7% (4.5%, 19.5%) | 13/62 = 21.0% (12.7%, 32.6%) |
|  | **GM (overall)** | 438 (240, 801) | 1426 (844, 2410) | 5.82 (3.22, 10.52) | 10.79 (5.52, 21.08) |
|  | **GM (among positive responders)** | 1334 (944, 1886) | 2528 (1754, 3643) | 607 (72, 5120) | 553 (233, 1311) |
| **IgG TV1c8.2.C V1V2** | **Response rate** | 36/57 = 63.2% (50.2%, 74.5%) | 49/57 = 86.0% (74.7%, 92.7%) | 6/57 = 10.5% (4.9%, 21.1%) | 9/57 = 15.8% ( 8.5%, 27.4%) |
|  | **GM (overall)** | 369 (142, 959) | 2252 (1250, 4057) | 13.61 (6.98, 26.54) | 16.32 (7.39, 36.04) |
|  | **GM (among positive responders)** | 3295 (2031, 5347) | 3835 (2585, 5690) | 611 (290, 1287) | 1245 (504, 3074) |
| **IgG CaseA2_gp70_V1V2.B** | **Response rate** | 39/65 = 60.0% (47.9%, 71.0%) | 53/65 = 81.5% (70.4%, 89.1%) | 6/62 = 9.7% (4.5%, 19.5%) | 11/62 = 17.7% (10.2%, 29.0%) |
|  | **GM (overall)** | 97.36 (47.75, 198.49) | 750 (390, 1442) | 4.26 (2.4, 7.56) | 5.94 (3.09, 11.41) |
|  | **GM (among positive responders)** | 629 (405, 977) | 1888 (1274, 2798) | 512 (226, 1157) | 568 (254, 1270) |
| **CD4+**  **Env ZM96.C** | **Response rate** | 34/56 = 60.7% (47.6%, 72.4%) | 41/56 = 73.2% (60.4%, 83.0%) | 23/55 = 41.8% (29.7%, 55.0%) | 35/55 = 63.6% (50.4%, 75.1%) |
|  | **GM (overall)** | 0.1163 (0.0918, 0.1474) | 0.1324 (0.1036, 0.1692) | 0.0735 (0.0563, 0.096) | 0.0934 (0.0724, 0.1204) |
|  | **GM (among positive responders)** | 0.1995 (0.1585, 0.251) | 0.1973 (0.1592, 0.2446) | 0.1925 (0.1416, 0.2616) | 0.1582 (0.1235, 0.2026) |
| **CD4+**  **Env 1086.C** | **Response rate** | 26/56 = 46.4% (34.0%, 59.3%) | 27/56 = 48.2% (35.7%, 61.0%) | 16/55 = 29.1% (18.8%, 42.1%) | 26/55 = 47.3% (34.7%, 60.2%) |
|  | **GM (overall)** | 0.085 (0.0684, 0.1057) | 0.082 (0.0644, 0.1046) | 0.054 (0.0427, 0.0681) | 0.0621 (0.0491, 0.0785) |
|  | **GM (among positive responders)** | 0.1722 (0.1383, 0.2145) | 0.1804 (0.1451, 0.2243) | 0.1633 (0.1161, 0.2297) | 0.1287 (0.0987, 0.1678) |
| **CD4+**  **Env TV1.C** | **Response rate** | 38/56 = 67.9% (54.8%, 78.6%) | 40/56 = 71.4% (58.5%, 81.6%) | 21/55 = 38.2% (26.5%, 51.4%) | 34/55 = 61.8% (48.6%, 73.5%) |
|  | **GM (overall)** | 0.119 (0.0944, 0.15) | 0.1331 (0.1061, 0.1669) | 0.0702 (0.0551, 0.0894) | 0.079 (0.0623, 0.1002) |
|  | **GM (among positive responders)** | 0.1794 (0.1428, 0.2253) | 0.197 (0.1605, 0.2418) | 0.1739 (0.1275, 0.2373) | 0.1329 (0.1043, 0.1693) |
