# Supplementary material for: Safety and immune responses after a 12-month booster in healthy HIV-uninfected adults in HVTN 100 in South Africa: A randomized double-blind placebo-controlled trial of ALVAC-HIV (vCP2438) and bivalent subtype C gp120/MF59 vaccines
Source: PLoS Med. 2020 Feb 24;17(2):e1003038. doi: 10.1371/journal.pmed.1003038 (PMC7039414; doi:10.1371/journal.pmed.1003038)
Supplement: S4 Table — Effector and central memory sub-populations are exploratory. (DOCX) [file pmed.1003038.s010.docx]

**S4 Table:** **Response rates (95% CIs) and geometric mean (GM) magnitudes (95% CIs) overall and among positive responders of secondary and exploratory humoral and cellular responses at peak (months 6.5, 12.5) and durability (months 12, 18) timepoints.** (Effector and central memory sub-populations are exploratory.)

| **Endpoint** | **Statistic** | **Peak Timepoints** | | **Durability Timepoints** | |
| --- | --- | --- | --- | --- | --- |
|  |  | **Month 6.5**  **Estimate**  **(95% CI)** | **Month 12.5**  **Estimate**  **(95% CI)** | **Month 12 Estimate**  **(95% CI)** | **Month 18 Estimate**  **(95% CI)** |
| **IgG3**  **ZM96.C**  **gp120** | **Response rate** | 28/65 = 43.1% (31.8%, 55.2%) | 37/65 = 56.9% (44.8%, 68.2%) | 3/64 = 4.7% (1.6%, 12.9%) | 3/64 = 4.7% (1.6%, 12.9%) |
|  | **GM**  **(overall)** | 69.99 (45.79, 106.98) | 120 (74, 196) | 7.42 (5, 11.01) | 6.08 (4.11, 8.99) |
|  | **GM (among positive responders)** | 305 (221, 421) | 443 (299, 657) | 419 (39, 4464) | 269 (30, 2459) |
| **IgG3**  **1086.C**  **gp120** | **Response rate** | 65/65 =100.0% (94.4%, 100.0%) | 58/65 = 89.2% (79.4%, 94.7%) | 19/64 = 29.7% (19.9%, 41.8%) | 17/64 = 26.6% (17.3%, 38.5%) |
|  | **GM**  **(overall)** | 2076 (1560, 2764) | 679 (439, 1051) | 40.02 (26.56, 60.29) | 36.69 (23.67, 56.86) |
|  | **GM (among positive responders)** | 2076 (1560, 2764) | 1011 (713, 1433) | 244 (177, 338) | 263 (169, 409) |
| **IgG3**  **TV1c8.2.C**  **gp120** | **Response rate** | 56/65 = 86.2% (75.7%, 92.5%) | 44/65 = 67.7% (55.6%, 77.8%) | 3/64 = 4.7% (1.6%, 12.9%) | 5/64 = 7.8% (3.4%, 17.0%) |
|  | **GM**  **(overall)** | 364 (246, 539) | 187 (119, 294) | 5 (3.36, 7.44) | 6.53 (4.1, 10.39) |
|  | **GM (among positive responders)** | 560 (428, 732) | 462 (334, 640) | 359 (30, 4326) | 333 (232, 478) |
| **IgG3**  **ZM96.C**  **V1V2** | **Response rate** | 5/64 = 7.8% (3.4%, 17.0%) | 5/64 = 7.8% (3.4%, 17.0%) | 1/63 = 1.6% (0.3%, 8.5%) | 0/63 = 0.0% (0.0%, 5.7%) |
|  | **GM**  **(overall)** | 6.46 (3.23, 12.92) | 6.87 (3.53, 13.38) | 2.47 (1.48, 4.1) | 2.66 (1.57, 4.5) |
|  | **GM (among positive responders)** | 1741 ( 190, 15975) | 2189 ( 341, 14064) | 886 (-, -) | NA |
| **IgG3 1086.C V1V2** | **Response rate** | 15/65 = 23.1% (14.5%, 34.6%) | 14/65 = 21.5% (13.3%, 33.0%) | 1/64 = 1.6% (0.3%, 8.3%) | 0/64 = 0.0% (0.0%, 5.7%) |
|  | **GM (overall)** | 14.86 (8.13, 27.17) | 15.82 (9.35, 26.78) | 2.11 (1.59, 2.8) | 1.84 (1.4, 2.43) |
|  | **GM (among positive responders)** | 490 (211, 1137) | 335 (174, 643) | 280 (-, -) | NA |
| **IgG3 TV1c8.2.C V1V2** | **Response rate** | 7/64 = 10.9% (5.4%, 20.9%) | 7/64 = 10.9% (5.4%, 20.9%) | 0/63 = 0.0% (0.0%, 5.7%) | 0/63 = 0.0% (0.0%, 5.7%) |
|  | **GM (overall)** | 4.25 (2.42, 7.46) | 5.1 (2.92, 8.92) | 1.29 (1.04, 1.61) | 1.29 (1.02, 1.62) |
|  | **GM (among positive responders)** | 571 (223, 1458) | 562 (178, 1768) | NA | NA |
| **IgG3 CaseA2_gp70_V1V2.B** | **Response rate** | 5/64 = 7.8% (3.4%, 17.0%) | 6/64 = 9.4% (4.4%, 19.0%) | 0/63 = 0.0% (0.0%, 5.7%) | 2/63 = 3.2% (0.9%, 10.9%) |
|  | **GM (overall)** | 2.41 (1.56, 3.72) | 3.19 (1.9, 5.33) | 1.1 (0.98, 1.24) | 1.24 (0.99, 1.56) |
|  | **GM (among positive responders)** | 350 (128, 956) | 396 (130, 1200) | NA | 117 (114, 121) |
| **nAb TV1c8.2.C** | **Response rate** | 67/69 = 97.1% (90.0%, 99.2%) | 68/69 = 98.6% (92.2%, 99.7%) | 10/60 = 16.7% (9.3%, 28.0%) | 54/60 = 90.0% (79.9%, 95.3%) |
|  | **GM (overall)** | 98.06 (76.9, 125.05) | 331 (248, 443) | 6.07 (5.4, 6.84) | 40.78 (30.55, 54.44) |
|  | **GM (among positive responders)** | 107 (86, 133) | 352 (270, 460) | 16.07 (12.57, 20.55) | 51.49 (40.11, 66.1) |
| **nAb MW965.26.C** | **Response rate** | 68/69 = 98.6% (92.2%, 99.7%) | 68/69 = 98.6% (92.2%, 99.7%) | 6/67 = 9.0% (4.2%, 18.2%) | 56/67 = 83.6% (72.9%, 90.6%) |
|  | **GM (overall)** | 272 (202, 367) | 774 (555, 1081) | 5.52 (5.07, 6) | 35.33 (25.24, 49.46) |
|  | **GM (among positive responders)** | 289 (219, 382) | 826 (604, 1129) | 14.96 (8.93, 25.07) | 51.87 (37.92, 70.95) |
| **CD4+**  **Effector Memory to Env ZM96.C** | **Response rate** | 37/67 = 55.2% (43.4%, 66.5%) | 44/68 = 64.7% (52.8%, 75.0%) | 21/65 = 32.3% (22.2%, 44.4%) | 36/67 = 53.7% (41.9%, 65.1%) |
|  | **GM (overall)** | 0.0705 (0.0563, 0.0884) | 0.0822 (0.066, 0.1024) | 0.0443 (0.0361, 0.0543) | 0.0495 (0.0406, 0.0605) |
|  | **GM (among positive responders)** | 0.1391 (0.1089, 0.1776) | 0.1335 (0.1061, 0.1679) | 0.1146 (0.0769, 0.1708) | 0.0851 (0.0653, 0.1109) |
| **CD4+**  **Central Memory to Env ZM96.C** | **Response rate** | 37/67 = 55.2% (43.4%, 66.5%) | 44/68 = 64.7% (52.8%, 75.0%) | 21/65 = 32.3% (22.2%, 44.4%) | 36/67 = 53.7% (41.9%, 65.1%) |
|  | **GM (overall)** | 0.0366 (0.0318, 0.0421) | 0.035 (0.0305, 0.0402) | 0.0324 (0.0286, 0.0367) | 0.0347 (0.0306, 0.0393) |
|  | **GM (among positive responders)** | 0.0482 (0.0386, 0.0603) | 0.0424 (0.0347, 0.0516) | 0.0507 (0.0383, 0.0672) | 0.0453 (0.0371, 0.0552) |
| **CD4+**  **Effector Memory to Env 1086.C** | **Response rate** | 31/67 = 46.3% (34.9%, 58.1%) | 33/68 = 48.5% (37.1%, 60.2%) | 12/65 = 18.5% (10.9%, 29.6%) | 22/67 = 32.8% (22.8%, 44.7%) |
|  | **GM (overall)** | 0.0562 (0.046, 0.0687) | 0.06 (0.0489, 0.0735) | 0.0349 (0.0299, 0.0409) | 0.037 (0.0314, 0.0436) |
|  | **GM (among positive responders)** | 0.1126 (0.086, 0.1474) | 0.1183 (0.0916, 0.1527) | 0.1208 (0.0823, 0.1772) | 0.0765 (0.0542, 0.1079) |
| **CD4+**  **Central Memory to Env 1086.C** | **Response rate** | 31/67 = 46.3% (34.9%, 58.1%) | 33/68 = 48.5% (37.1%, 60.2%) | 12/65 = 18.5% (10.9%, 29.6%) | 22/67 = 32.8% (22.8%, 44.7%) |
|  | **GM (overall)** | 0.0297 (0.0271, 0.0326) | 0.028 (0.026, 0.0302) | 0.0281 (0.0259, 0.0305) | 0.0287 (0.0262, 0.0315) |
|  | **GM (among positive responders)** | 0.0353 (0.0294, 0.0425) | 0.0314 (0.0272, 0.0364) | 0.0407 (0.0283, 0.0586) | 0.0381 (0.0294, 0.0494) |
| **CD4+**  **Effector Memory to Env TV1.C** | **Response rate** | 36/67 = 53.7% (41.9%, 65.1%) | 41/68 = 60.3% (48.4%, 71.1%) | 18/65 = 27.7% (18.3%, 39.6%) | 35/67 = 52.2% (40.5%, 63.7%) |
|  | **GM (overall)** | 0.0716 (0.0573, 0.0894) | 0.084 (0.0679, 0.104) | 0.0411 (0.0342, 0.0494) | 0.0425 (0.0355, 0.0508) |
|  | **GM (among positive responders)** | 0.1437 (0.1126, 0.1834) | 0.1437 (0.115, 0.1796) | 0.1104 (0.0748, 0.1631) | 0.0672 (0.0512, 0.0881) |
| **CD4+**  **Central Memory to Env TV1.C** | **Response rate** | 36/67 = 53.7% (41.9%, 65.1%) | 41/68 = 60.3% (48.4%, 71.1%) | 18/65 = 27.7% (18.3%, 39.6%) | 35/67 = 52.2% (40.5%, 63.7%) |
|  | **GM (overall)** | 0.0327 (0.029, 0.037) | 0.0308 (0.0279, 0.034) | 0.0309 (0.0277, 0.0344) | 0.0306 (0.0278, 0.0337) |
|  | **GM (among positive responders)** | 0.0413 (0.0335, 0.051) | 0.0355 (0.0305, 0.0414) | 0.049 (0.0369, 0.065) | 0.0363 (0.0307, 0.043) |
| **ADCP**  **1086.C gp140** | **Response rate** | 60/60 =100.0% (94.0%, 100.0%) | 60/60 =100.0% (94.0%, 100.0%) | 27/57 = 47.4% (35.0%, 60.1%) | 47/57 = 82.5% (70.6%, 90.2%) |
|  | **GM (overall)** | 11.26 (10.72, 11.83) | 11.21 (10.35, 12.15) | 4.18 (3.5, 4.99) | 7.13 (6.1, 8.35) |
|  | **GM (among positive responders)** | 11.26 (10.72, 11.83) | 11.21 (10.35, 12.15) | 6.91 (6.13, 7.78) | 8.76 (8.01, 9.57) |
